# Low-dose rapamycin alleviates clinical symptoms of fatigue and PEM in ME/CFS patients via improvement of autophagy: a pilot study

**DOI:** 10.1186/s12967-025-07213-8

**Published:** 2025-10-21

**Authors:** Brian T. Ruan, Sarojini Bulbule, Brooke Gile, Amy Reyes, Bela Chheda, Lucinda Bateman, Jennifer Bell, Brayden Yellman, Stephanie L. Grach, Jon Berner, Daniel L. Peterson, David Kaufman, Avik Roy, C. Gunnar Gottschalk

**Affiliations:** 1 Cornell EIND Center, Cornell University, Ithaca, NY USA; 2 Research and Development Lab, Simmaron Research Inc., University of Wisconsin-Milwaukee, Milwaukee, USA; 3 Center for Complex Diseases, Palo Alto, CA USA; 4 Bateman Horne Center, Salt Lake City, UT USA; 5 Mayo Clinic, Rochester, MN USA; 6 Woodinville Psychiatry, Woodinville, WA USA; 7 Sierra Internal Medicine, Incline Village, NV USA; 8 Simmaron Research Center for Translational Science, ICBI at Indiana University Methodist Hospital, Indianapolis, IN USA; 9 Milwaukee Institute for Drug Discovery, University of Wisconsin- Milwaukee, 2000 E Kenwood Blvd, Milwaukee, WI 53211 USA; 10 Center for Complex Diseases, Seattle, WA USA

## Abstract

**Background:**

mTOR activation is associated with chronic inflammation in ME/CFS. Previous studies have shown that sustained mTOR activation may cause chronic muscle fatigue by inhibiting ATG13-mediated autophagy. However, the therapeutic implication of this finding has not been established. Given that rapamycin is an mTOR inhibitor, this study aims to investigate whether low-dose rapamycin treatment improves autophagy markers and clinical symptoms of fatigue in ME/CFS subjects. This highlights the pivotal role of mTOR in the pathogenesis of ME/CFS.

**Methods:**

We conducted a decentralized, uncontrolled trial of rapamycin in 86 patients with ME/CFS to evaluate its safety and efficacy. Low-dose rapamycin (6 mg/week) was administered, and core ME/CFS symptoms were assessed on days 30 (T1), 60 (T2), and 90 (T3). Plasma levels of autophagy metabolites, such as pSer258-ATG13 and BECLIN-1, were measured and correlated with clinical outcomes, specifically MFI.

**Results:**

Rapamycin (6 mg/week) was tolerated without any SAEs. Of the 70 patients who completed at the minimum to T1, 52 (74.3%) showed recovery in fatigue, PEM, and OI, along with improvements in MFI fatigue domains and SF-36 aspects. High levels of BECLIN-1 were detected in T3. Plasma pSer258-ATG13 levels were strongly downregulated at T1. Spearman’s correlation analysis indicated an association between autophagy impairment and reduced activity.

**Conclusions:**

Low-dose rapamycin effectively reduced PEM and other key symptoms in patients with ME/CFS, as measured by BAS, SSS, MFI, and SF-36. Future studies should encompass dose optimization and develop a diagnostic tool to identify responders with mTOR-mediated autophagy disruption.

**Graphical Abstract:**

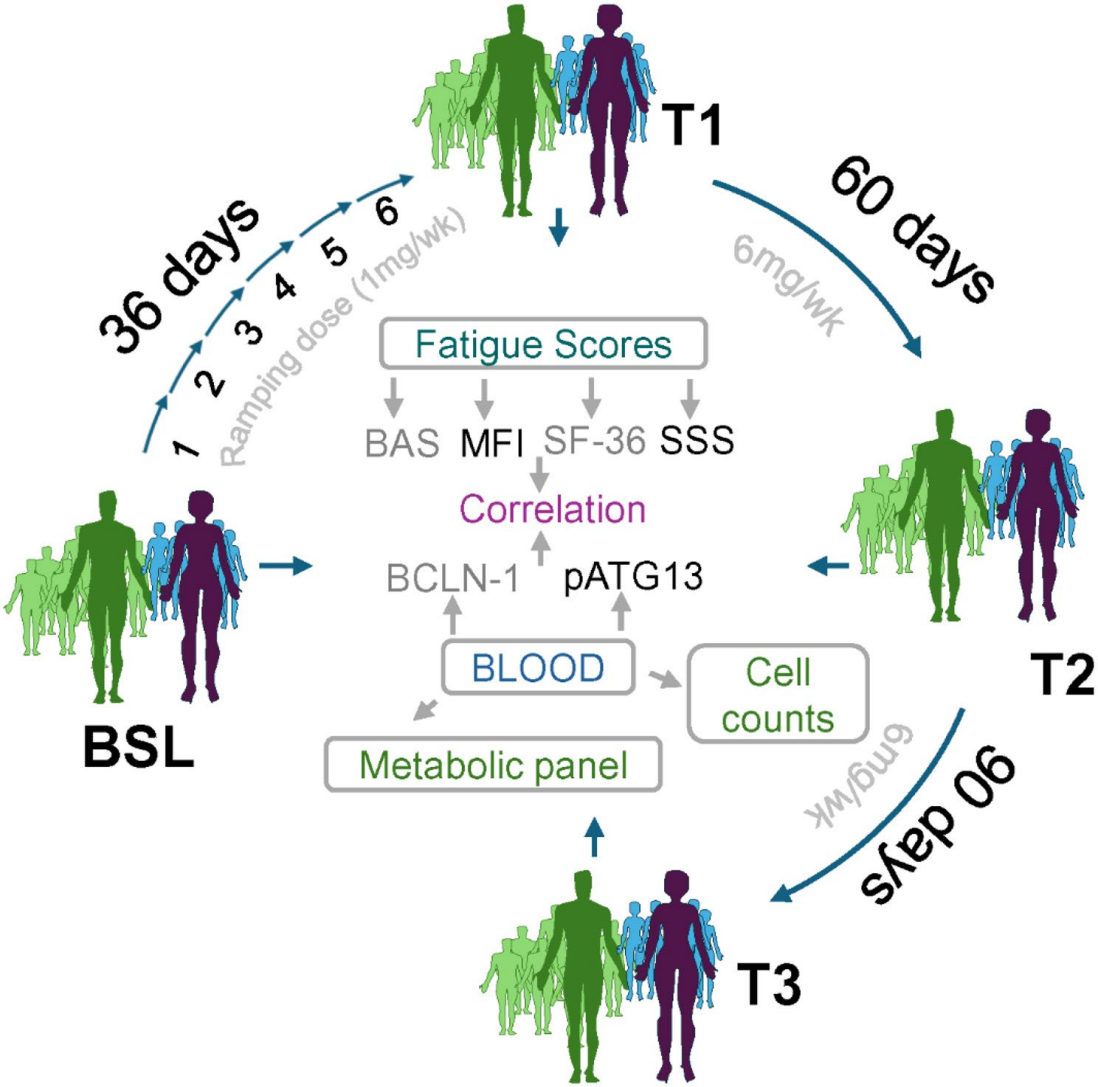

**Supplementary Information:**

The online version contains supplementary material available at 10.1186/s12967-025-07213-8.

## Highlights

The first decentralized biomarker-directed clinical trial of rapamycin, an mTOR inhibitor, was used to treat ME/CFS.

The correlation between autophagy markers such as BECLIN-1 and pSer258-ATG13 and hallmark symptoms of ME/CFS patients with post-exertional malaise.

Validation of phosphorylated ATG13 (Ser258) in plasma samples and exploration of its correlation with key disease-defining symptoms in ME/CFS patients.

**Supplementary Information:**

The online version contains supplementary material available at 10.1186/s12967-025-07213-8.

## Introduction

Myalgic encephalomyelitis/chronic fatigue syndrome (ME/CFS) is a complex, multisystem disorder characterized by profound fatigue, post-exertional malaise (PEM), musculoskeletal pain, cognitive dysfunction, and orthostatic intolerance (OI) [1, 2]. An estimated 3 million individuals in the United States are affected by ME/CFS [3]. Diagnostic criteria established by the Institute of Medicine and the 2003 Canadian Consensus Criteria emphasize PEM as the cardinal feature distinguishing ME/CFS from other clinically similar conditions [4, 5]. PEM is defined as a delayed and prolonged exacerbation of ME/CFS symptoms, including severe fatigue, myalgia, light and sound sensitivity, flu-like symptoms, and cognitive dysfunction following minimal physical or mental exertion. These episodes are often incapacitating, typically lasting more than 72 h, and in some cases persisting for weeks or longer. Evidence suggests that recurrent or overlapping PEM episodes may be additive and potentially contribute to irreversible physiological deterioration, underscoring the need for effective therapeutic intervention. OI, another hallmark of ME/CFS [6], is characterized by abnormal cardiovascular responses to an upright posture, including hypotension and tachycardia. Despite substantial research efforts, the pathophysiology of ME/CFS remains unclear. Emerging evidence implicates abnormalities in several metabolic and cellular pathways, including impaired glycolysis [7, 8], reduced mitochondrial oxygen consumption [9], deficits in energy metabolism [10], and disrupted autophagy [11] as potential contributors to disease pathogenesis.

Currently, no FDA-approved therapies are available for the treatment of ME/CFS. Symptomatic treatments—used off-label to manage key features such as post-exertional malaise (PEM) and orthostatic intolerance (OI)—offer limited benefit and lack a well-defined mechanism of action. The absence of mechanistic clarity makes it challenging to predict therapeutic responses in a highly heterogeneous patient population.

Our previous study [11] demonstrated that serum samples from ME/CFS subjects exhibited elevated levels of the early autophagy protein, ATG13. Further analysis revealed that the elevated ATG13 in these serum samples was heavily phosphorylated at its serine residue, rendering it non-functional and potentially indicative of impaired autophagy. Accordingly, in a mouse model [12], genetic ablation of ATG13 resulted in severe muscle fatigue following treadmill exercise, suggesting a crucial role for ATG13 in the pathogenesis of ME/CFS. The mammalian target of rapamycin (mTOR) is the upstream kinase [13] responsible for phosphorylating ATG13 [14] at its Ser258 residue [15] during starvation-induced autophagy impairment. Notably, our study [12] found that the pharmacological activation of mTOR through oral administration of the mTOR agonist MHY1485 resulted in serine phosphorylation at ATG13 and impaired macroautophagy by dislodging ATG13 from the early autophagy complex, leading to mononucleosis and increased infiltration of inflammatory macrophages in the vasculature of muscle tissue, which caused demyelination of nerves serving muscles [12] Other studies [16, 17] have also reported that the activation of mTOR in monocytes induces pro-inflammatory responses in chronic cardiovascular diseases. These studies strongly suggest that inactivation of mTOR may be beneficial in alleviating the pathological symptoms of chronic inflammatory diseases. To investigate the role of mTOR inhibition in alleviating clinical symptoms of ME/CFS, we initiated an observational trial with low-dose, once weekly (6 mg/wk) Rapamycin (*sirolimus*), an mTOR inhibitor, in ME/CFS subjects.

Rapamycin, a selective mTOR inhibitor [18, 19], was approved by the FDA in 1999 [20] as an immunosuppressant to prevent organ rejection and later in 2003 for use in cardiac stents to prevent restenosis [21]. It is actively researched and used off-label for various cancers [22, 23], including tuberous sclerosis [24], acute myeloid leukemia [25], and cancers of renal [26], breast [27], and lung [28] origins. Its safety profile has been well studied [29]. Rapamycin has been recently investigated as a geroprotective drug [30]. A study conducted [30] by the Interventions Testing Program (ITP), a peer-reviewed NIH program, along with other research [31], has led to ongoing trials of rapamycin for longevity in humans^33^ as well as its widespread off-label use for geroprotection [32].

Low-dose rapamycin (6 mg/week) was administered to 86 individuals with ME/CFS, of whom 40 completed the full 90-day study protocol. As of the submission of this manuscript, 6 patients remain on the study protocol. The study included four time points for clinical and biospecimen data collection: a pretreatment baseline (BSL; day 0), followed by longitudinal assessments at day 36 (T1), day 60 (T2), and day 90 (T3), while participants remained on therapy. At each time point, peripheral blood was collected via mobile phlebotomy, and samples were processed and analyzed at the Simmaron-UWM Laboratory. Safety laboratory assessments were performed concurrently using LabCorp^®^. Clinical endpoints were assessed using self-reported validated instruments endorsed by the NIH Common Data Elements for ME/CFS, including measures of fatigue, post-exertional malaise (PEM), orthostatic intolerance (OI), and related symptoms. Biological assays focused on quantifying autophagy-associated proteins, including BECLIN-1 and phosphorylated ATG13 at serine 258 (pSer258-ATG13), at each time point. Nonparametric correlation analyses demonstrated significant clinical improvement in 38 of the 40 participants who completed the 90-day study, characterized by reductions in fatigue, PEM, and OI. These clinical responses were accompanied by the upregulation of BECLIN-1 and suppression of pSer258-ATG13, supporting a mechanistic link between the therapeutic benefit and restoration of autophagy signaling.

## Materials and methods

### Trial design, IRB approval, and oversight

This rapamycin trial in ME/CFS was a non-controlled, decentralized, observational clinical study conducted across six clinical centers in the United States. Participants were recruited and remained under the care of site-specific principal investigators at the following institutions: The Center for Complex Diseases (Bela Chheda, MD, Mountain View, CA, and David Kaufman, MD, Seattle, WA), the Bateman Horne Center (Jennifer Bell, FNP, Salt Lake City, UT), Simmaron Research Clinical Center (Daniel Peterson, MD, Incline Village, NV), Mayo Clinic (Stephanie Grach, MD, Rochester, MN), and Woodinville Psychiatric (Jon Berner, MD, PhD, Woodinville, WA). All patients met the 2003 Canadian Consensus and the Institute of Medicine (IOM) clinical criteria. The study was conducted under a central protocol (SRI-RP-2023-1), and all sites were approved by the Western Institutional Review Board (WIRB Study Number: 1361805). Simmaron Research Inc. is the study sponsor, and the trial was registered on ClinicalTrials.gov (NCT06257420).

### Selection of patient cohort and other general information

Participants (*N* = 109) were enrolled in this trial by invitation only. Each participant was pre-screened by the site principal investigators to confirm that they met the clinical diagnostic criteria for ME/CFS based on the 2003 Canadian Consensus and IOM criteria. Written informed consent was obtained from all participants or their legal guardians before enrollment. Once enrolled, participants were continuously monitored by physicians at each clinical site.

### Safety lab and study sample collection

Upon completion of the baseline questionnaires, a certified mobile phlebotomy provider was scheduled for each enrolled participant. Four study sample kits (BSL-T3) were shipped to the participants’ residences before the scheduled blood collection. Each kit contained the following BD Vacutainer^®^ tubes (Franklin, NJ, USA): two 10 mL K2EDTA tubes, one 10 mL SST tube, one 4 mL sodium citrate (NaCit) tube, and one RNA PaxGene tube. Concurrent with the study sample collection, safety laboratory tests were performed using the same phlebotomy visit and submitted to LabCorp^®^ for analysis. Safety labs included the following assays: Comprehensive Metabolic Panel (CMP-14, #322000), Complete Blood Count with Differential (CBC-Diff, #005009), Lipid Panel (#303756), High-Sensitivity C-Reactive Protein (HS-CRP, #120766), and Hemoglobin A1C (#001453). Ordering physicians monitored the results. All samples were processed under Good Laboratory Practice (GLP) conditions within 12 h of collection at the Simmaron Research and Development Laboratory (University of Wisconsin–Milwaukee). Plasma, serum, and PaxGene RNA were extracted from whole blood, aliquoted immediately, and stored at − 80 °C or in liquid nitrogen. Comprehensive demographic and clinical data, including age, sex, ethnicity, body mass index (BMI), and disease duration, were recorded at enrollment and at each time point thereafter.

### Digital database maintenance in REDCap

The REDCap database used in this study was approved, housed, and managed at the Weill Cornell School of Medicine REDCap server in collaboration with the Cornell EIND Center [33, 34]. Electronic versions of all consent forms and study questionnaires were housed in the REDCap database.

### Study questionnaires (BSL-T3)

After resigning the electronic site-specific informed consent document, the patient completed the BSL questionnaires (electronically), which included the Medical Outcomes Study-36 Item Short Form-Health Survey (SF-36), Multidimensional Fatigue Inventory (MFI), Bell Activity Scale (BAS), Sleep Questionnaire, Specific Symptom Severity (SSS) Inventory, Medication List, Past and Current Illnesses (BSL only), Questionnaires for ME/CFS Patients (BSL only), and the 24-Hour-Health-Inventory. Follow-up questionnaires were distributed via REDCap at each time point, in line with the study protocol.

### The measurement protocol of the BAS, SSS, MFI, and SF-36

All participants were asked to complete a series of validated questionnaires and surveys to gauge the complexity and severity of symptoms over time. The primary study endpoints included BAS, SSS, MFI, and SF-36 scores. The BAS ranges from 0 to 100, where 0 indicates severe symptoms on a continuous basis, and 100 indicates no symptoms at rest or with exercise [35]. Participants also completed the SSS instrument, reporting the severity of common ME/CFS symptoms on a scale of 0 to 10, where 0 indicates not experienced and 10 indicates very severe symptoms. This inventory includes four hallmark symptoms of ME/CFS: fatigue, disturbed sleep, post-exertional malaise, and orthostatic intolerance. The MFI consists of 20 questions on a scale of 1 to 5, quantifying fatigue in five different domains [36]. Each domain consists of four items with scores between 4 and 20, where 4 indicates low to no fatigue and 20 indicates severe fatigue. The patients also completed the RAND 36-Item Short Form Health Survey (SF-36, version 1), which quantifies various quality of life measures [37]. Scores were scaled to the mean of the healthy US population according to the scoring instructions [38].

### Stratification based on BAS, MFI, and SF-36 criteria for responder analysis

The current literature is limited compared to responder analysis in ME/CFS clinical trials. However, previous studies [39–41] have examined empirical methods to identify patients with ME/CFS using the SF-36. The most relevant subscales were VT, SF, and RP. Another study [42] included the PCS aggregate score in their responder analysis of patients with anemia and chronic kidney disease. They also identified a three-point differential in the SF-36 subscales and a two-point differential in the PCS as the threshold for a minimal clinically important difference (MCID) between time points. Accordingly, the ME/CFS patients in this rapamycin trial were stratified as follows.

SF-36

- Responder: Two or more of the following:
  - ≥ +3 in VT sub-scale.
  - ≥ +3 in SF sub-scale.
  - ≥ +3 in RP sub-scale.
  - ≥ +2 in PCS sub-scale.
- Partial responder: One of the above.
- Non-responder: None of the above.

Stroke rehabilitation studies [43] have proposed a -5 to -7.33-point differential as the MCID for the MFI aggregate score. For ME/CFS, a minus two-point differential was established as the MCID for each MFI fatigue domain [44]. An extrapolation to the MFI aggregate would suggest that a minus-ten-point differential is required as the MCID for the MFI aggregate score. Accordingly, participants were stratified as follows:

MFI

- Responder:
  - ≤ -2 for three or more of the five fatigue domains.
  - ≤ -2 in two of the five domains AND ≤ -10 in the MFI aggregate.
- Partial responder:
  - ≤ -2 for two of the five fatigue domains.
  - ≤ -2 in one of the five domains and − 10 < MFI aggregate < 0.
- Non-responder: None of the above.

Although the BAS is frequently employed in ME/CFS studies [45] to quantify functional ability, its use as a formal responder classification tool remains limited. Thus, we propose a new methodology for future responder analysis.

BAS

- Responder: ≥ +10 in BAS.
- Non-responder: ≤ 0 in BAS.

The partial responder stratum was excluded because the BAS operates at intervals of ten, and a ten-point differential in the BAS is descriptively significant. Furthermore, a theoretical twenty-point differential for the responder subset to accommodate a partial responder subset would be too conservative for a stratification approach.

Finally, to classify participants, their overall responder status was determined as such:

- Responder: ≥ 2 questionnaires (SF-36, MFI, or BAS) reported as “responder”.
- Partial responder: 1 questionnaire (SF-36, MFI, or BAS) reported as “partial responder,” but not a “responder”.
- Non-responder: None of the above.

Differentials were measured between baseline and timepoint 3 for each participant.

### Exploratory training of a machine learning model to classify non-completer participants using multinomial logistic regression

Data were first preprocessed to include only the features of interest: BAS, MFI (all), SF-36 RP, VT, SF subscales, and the PCS aggregate score. Missingness tables were constructed to assess robust imputation methods. Because there were missing data, multiple imputation by chained equations (MICE) was performed using the IterativeImputer class using up to 10 iterations, as well as k-Nearest Neighbors (kNN) with k = 5 and k = 10 among completers and non-completers together. MICE was selected based on similarity and robustness of imputed values. Differentials were measured between baseline and timepoint 3. Subsequent analysis focused on the completer cohort, stratified using the decision tree classifier above. The pipeline included the use of StandardScaler to normalize feature values prior to fitting a multinomial logistic regression model. The model was implemented with an L2 penalty (Ridge), L-BFGS solver, and a maximum of 1,000 fitting iterations. Performance was evaluated using repeated stratified k-fold cross-validation, and the final approach used 3 splits and 5 repeats. Model coefficients with 95% confidence intervals and odds ratios were computed for interpretability. To address observed instability in odds ratios, L1 regularization was applied to identify key features in a two-step approach. The refined model was re-evaluated with repeated stratified cross-validation, classification report metrics, confusion matrix, and calibration curve. Finally, the refined model was applied to the non-completer cohort to assess generalizability. This workflow required the use of Python version 3.12.8 with the scikit-learn package version 1.7.1.

### Study events

Safety laboratory assessments, study sample collection, and REDCap questionnaire administration were conducted according to a standardized study timeline. Assessments were performed at the following time points: baseline (within two weeks prior to initiating rapamycin treatment), time point 1 (T1; following a six-week dose escalation from 1 mg/week to 6 mg/week), time point 2 (T2; 30 days after T1), and time point 3 (T3; 30 days after T2). Additional optional follow-up assessments (T3–T7) were conducted every 90 days following T3 using the same schedule of events. Upon initiation of the first rapamycin dose, the study team scheduled subsequent study events to ensure adherence to the protocol timeline. Study drug adherence was monitored by checking physician-derived pharmacy fill and refill records. Adherence was monitored by checking physician-derived prescription telemedicine and/or in-person office visits with their enrolling clinician (clinical PI). Patients communicated any AEs, SAEs, dose changes, or dose stoppages directly to their clinicians and the overall study PI. These findings have been reported and documented previously.

### Obtaining study drug

After reviewing the baseline safety laboratories and monitoring drug-drug interactions, study physicians prescribed the study drug, which the patients filled at their local pharmacy of choice. Study drug compliance was measured by pharmacy drug fill/refill receipt.

### Biobanking of serum, plasma, and PBMCs

Upon receipt, plasma samples were aliquoted and immediately stored in a -80^◦^C freezer located at suite #353 of the Milwaukee Institute for Drug Discovery (MIDD). PBMCs were isolated using an optimized magnetic separation protocol developed by Stem Cell Technologies (Cambridge, MA), counted using an automated digital counter (Invitrogen Countess, Invitrogen, Waltham, MA), and then stored in a liquid nitrogen storage tank (ThermoFisher INC) at MIDD.

### ELISA analysis of BECLIN-1

ELISA of BECLIN-1 was performed based on a kit (Cat# ab254511) and by a protocol described in the manufacturer’s protocol.

### Development of ATG13 ELISA and analysis in plasma samples

The Ser258 phospho-specific ATG13 antibody was developed for this study in collaboration with Thermo Fisher Scientific’s custom antibody synthesis division. The standard immunization protocol was adopted by injecting peptide antigen (0.5 mg) into rabbits and collecting serum samples 28, 56, and 72 days after the first immunization. The crude sera were affinity-purified, validated by ELISA with serial dilution, and provided to our laboratory. For ELISA, Nunc MicroWell 96-Well Optical-Bottom Plates with polystyrene Base (Cat# 165305; ThermoFisher) were used. Briefly, the plasma samples (or PBMC lysates) were mixed with carbonate coating buffer (Cat# CB01100; ThermoFisher Scientific; mixed with 25 µM of Na3VO4 and 1 µM of protease inhibitor cocktail) at a dilution of 1:2 (v/v), followed by addition to the plate overnight at 4 °C. After the coating process, the plate was blocked with 2% goat serum (Cat# 01-6201; ThermoFisher) for 30 min at room temperature and then treated with our custom-made pSer258-ATG13 antibody at a dilution of 1:100 with TBS-based antibody diluent (Li-cor Biosciences). After 2 h of incubation, the plate was washed with 1X TBST for three times followed by incubation with biotin-conjugated goat anti-rabbit IgG (H + L) (Cat# A16100) for 1 h. After that, 3X washes with 1XTBST were performed, streptavidin-HRP conjugate (Cat# S911; ThermoFisher) was added for 30 min, and the color was finally developed with the “SuperSignal™ ELISA Femto Substrate (Cat# 37074; ThermoFisher)” solution. The color development was stopped by 0.1 N hydrochloric acid (stop solution) before saturation. The results were validated by conducting the same assay at least three times, each by a different researcher.

### Statistical analysis

**Fig. 1:**
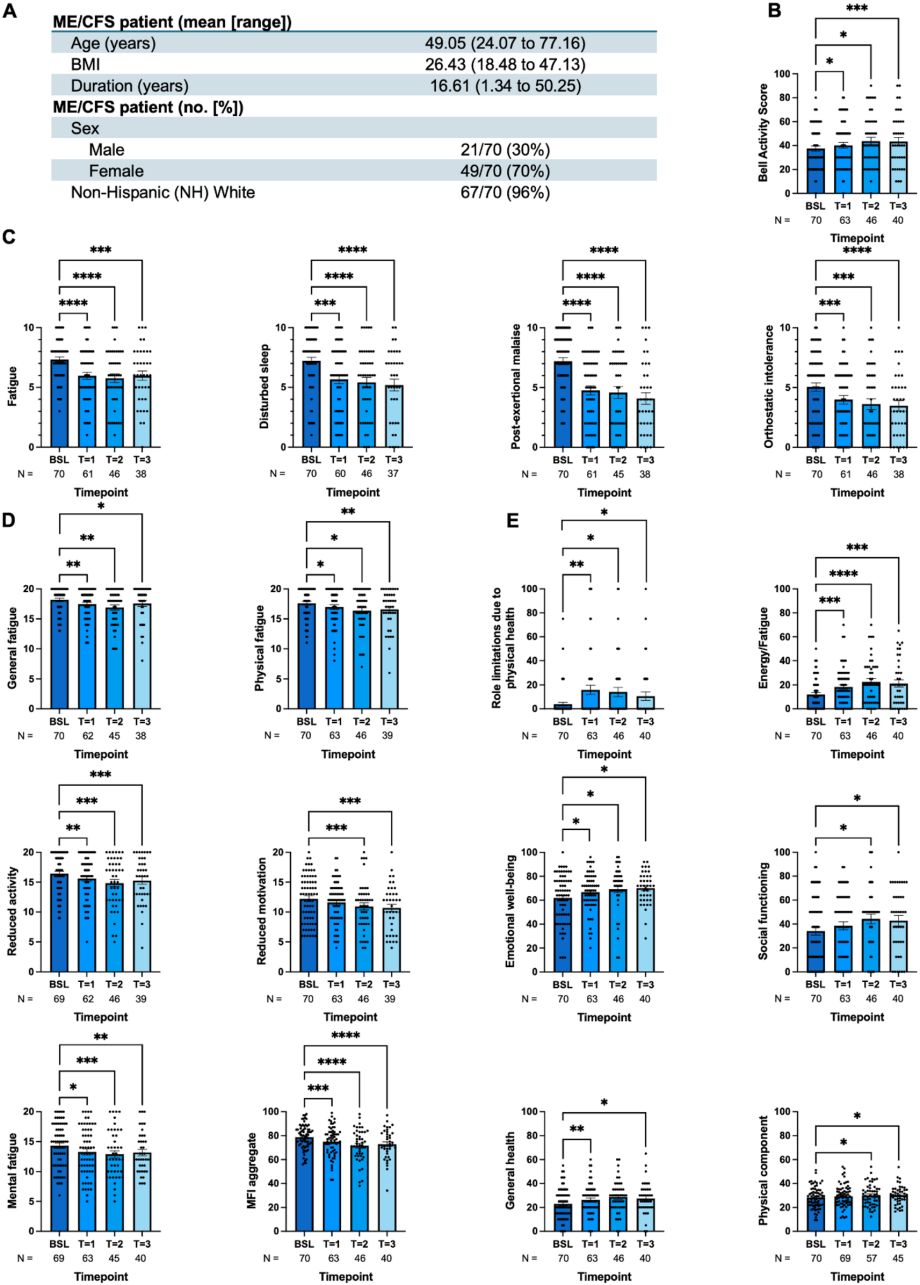
Low-dose rapamycin therapy significantly reduces clinical symptoms of fatigue in ME/CFS subjects. (**A**) The table summarized general information of the ME/CFS cohort (*N* = 70). Bar charts shown for the (**B**) Bell Activity Scale (BAS) and (**C**) Specific Symptom Scale (SSS) inventory of fatigue, disturbed sleep, PEM, and OI. Each point represents the sample of interest. (**D**) Different measurement criteria of Multidimensional Fatigue Inventory (MFI) including general fatigue, physical fatigue, reduced activity, reduced motivation, mental fatigue, and MFI aggregate were summarized in bar charts with individual scatter points annotated with sample size and timelines at bottom. (**E**) RAND 36-Item Short Form (SF-36) were analyzed based on role limitations due to physical health, emotional problems, energy/fatigue, emotional well-being, social functioning, and general health. Comparisons are from a mixed-effects model. The choice of statistical analysis and the details, including general fatigue, physical fatigue, reduced activity, reduced motivation, mental fatigue, and MFI aggregate, were summarized in bar charts with individual scatter points annotated with sample size and timelines discussed in method and result sections. Error bars are ± SEM. ^****^: *q* < 0.0001, ^***^: *q* < 0.001, ^**^: *q* < 0.01, ^*^: *q* < 0.05 (*q* = FDR-adjusted p values)

Demographic data were collected and are summarized in Fig. 1. The mean, range, and p-values from Student’s t-test were calculated for continuous variables. Percentages and p-values from the chi-square test of independence were calculated for categorical variables. Participant summary characteristics were prepared using pandas version 2.2.3 and SciPy version 1.15.1, and Python version 3.11.11.

**Fig. 2:**
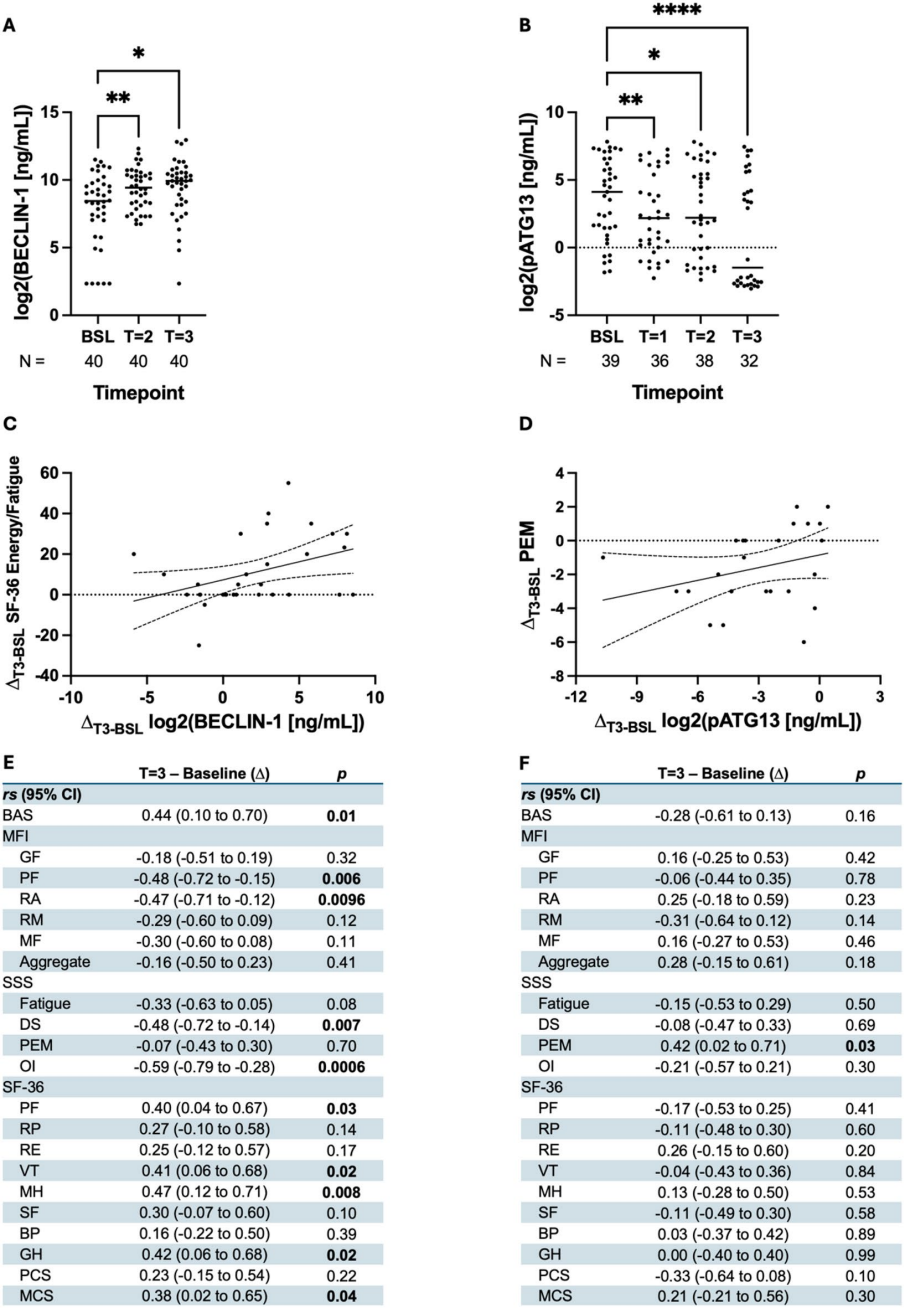
Low-dose rapamycin therapy significantly associated with increased autophagy marker BECLIN-1 and reduced pSer258-ATG13 in ME/CFS subjects. (**A**) BECLIN-1 and (**B**) pSer258-ATG13 ELISA was performed in plasma samples, converted to a base-2 logarithmic scale, and then plotted as a dot plot for different milestones. Sample size was included at the bottom of each timepoint, with comparisons made from a mixed-effects model. The correlation curve of the baseline to timepoint 3 difference of (**C**) SF-36 energy/fatigue sub-score vs. log2-BECLIN1—a significant (^*^p = 0.02) relationship—and (**D**) PEM vs. log2-pSer258-ATG13—a significant (^*^p = 0.03) relationship. Spearman coefficients (*rs*) for (**E**) BECLIN-1 and (**F**) pSer258-ATG13 for each questionnaire (BAS, MFI, SSS, and SF-36) are summarized in a table. ^****^: *p/q* < 0.0001, ^***^: *p/q* < 0.001, ^**^:*p/q* < 0.01, ^*^: *p*/*q* < 0.05. P and q-values were calculated while comparing groups for BECLIN-1 and pS258P-ATG13 respectively

Variations over time were plotted using column graphs (except for log2 biomarkers) with individual points and SEM bars. Plasma concentrations of BECLIN-1 and pSer258-ATG13 were log2-transformed. A mixed effects model was fit using restricted maximum likelihood (REML) to produce p-values, similar to a repeated measures ANOVA. Geisser-Greenhouse correction was used to correct for the assumption of sphericity in all variance analyses. Simple effects within participants comparing each time point to the baseline were corrected for multiple comparisons using Dunnett’s method. Subsequently, to control for the false discovery rate, the two-stage step-up method of Benjamini, Krieger, and Yekutieli (BKY FDR, α = 0.05) was applied to each analysis (interaction effects and multiple comparisons) to achieve global correction. Statistical significance was determined based on the resulting FDR-adjusted p-values (q-values). QQ plots were plotted for each mixed-effect model to test the normality of the residuals (Supplementary Fig. 1). Bivariate analysis was conducted using Spearman’s correlation and simple linear regression of the differences between baseline and timepoint 3 of log2(BECLIN-1) and log2(pSer258-ATG13) versus all questionnaires. The sample size was estimated based on typical Cohen’s D formula [46] for clinical pharm trials using a power = 0.8, effect size = 0.3, and an alpha of 0.05, which was calculated to be a minimum of 32 per timepoint. Statistical significance was set at *p* or *q* less than 0.05. Statistical analyses were performed using GraphPad Prism 10 for macOS version 10.6.0.

## Results

The dosing scheme had four milestones: baseline (BSL), timepoint 1 (T1), timepoint 2 (T2), and timepoint 3 (T3) phases (Supplement Fig. 2A). The T1 phase was designed for a duration of 36 days, commencing at a dosage of 1 mg/week and incrementally increasing by 1 mg per week, reaching 6 mg/week by the end of 36 days. This incremental adjustment was essential to account for the diverse drug responses among participants and to standardize all subjects to a uniform reference point. After T1, T2 phase (6 mg/wk) was continued until day 60 and T3 (6 mg/wk) phase till day 90. On the recruitment day, all subjects provided their consent, and their medical history was obtained (Supplement Fig. 2B). The BAS, which measures daily mobility and activity, was recorded. Other activity scales, such as the Pittsburgh Sleep Quality Index, MFI-20, and SF-36, were monitored throughout the study at each milestone as discussed in method section. Their medical history was obtained on the day of recruitment. Chemistry and metabolic panels, including HA1c, HS-CRP, and lipid profile, were recorded at each milestone (Supplement Fig. 2B).

A total of 109 participants were enrolled in this study between November 2023 and February 2025 (Supplement Fig. 3). Following the completion of baseline questionnaires and biospecimen collection, 23 individuals elected not to initiate study drug treatment after consultation with their respective clinical investigators. The remaining 86 participants were initiated on therapy with low-dose rapamycin. Of these, 70 completed the T1 phase (day 36), 55 completed the T2 phase (day 60), and 40 completed the full 90-day study protocol (T3), as shown in the study flowchart (Supplementary Fig. 3). 6 remained on the study protocol at the time of this manuscript’s submission.

Overall, the weekly low-dose rapamycin regimen was well tolerated, with minimal adverse events (AEs) reported (Table 1). The most common AEs, transient gastrointestinal symptoms, headache, and insomnia, were primarily observed during the baseline period and declined over time, suggesting adaptation to therapy. Importantly, discontinuation from the study was most commonly attributed to financial barriers as this pilot trial did not cover the cost of the study drug or safety laboratory tests. A secondary reason for discontinuation was a lack of perceived clinical benefit or a clinical decision to initiate a different therapy that may or may not interact with the study protocol.

**Table 1:** Treatment stops at each timepoint

	RAMP Period	T1 to T2	T2 to T3
**Adverse Events/Reason**			
Diarrhea/GI complaints	5	2	0
Insomnia	3	3	0
Headache	0	1	0
No change in symptoms	0	8	9
Financially unable	8	2	0
**Total**	16	15	9

Provided were the reasons why participants decided to stop rapamycin therapy

*Rapamycin provides significant relief from key clinical symptoms in ME/CFS patients as measured by the BAS*,* SSS*,* MFI and SF-36.*

The demographic and clinical characteristics of the completer subgroup (*N* = 70), including age, sex, BMI, ethnicity, and disease duration, are summarized in Fig. 1A.

### Bell activity scale

Treatment with low-dose rapamycin led to a statistically significant improvement in physical activity levels, as measured by the Bell Activity Scale BAS (Fig. 1B). Mixed model analysis revealed a significant effect of time (*F*_2.33, 114_ = 8.36, ^****^q < 0.0001), indicating progressive improvement across the 90-day treatment period. Post-hoc pairwise comparisons using Dunnett’s multiple comparisons test showed a significant 14.99% increase in BAS scores, from a mean of 37.4 at baseline to 43.3 at timepoint 3 (^***^q = 0.0002). These findings indicate that clinically meaningful improvements in physical activity emerged gradually over the course of rapamycin therapy, with the most robust effects observed after 90 days of continuous treatment.

### Specific symptom severity inventory

Low-dose rapamycin treatment resulted in a significant improvement in several core ME/CFS symptoms, as measured by patient-reported outcomes on the Symptom Severity Scale (Fig. 1C). Mixed effects modeling showed statistically significant reductions in fatigue (*F*_2.92, 138_ = 15.8, ^****^q < 0.0001), disturbed sleep (*F*_2.66, 124_ = 13.7, ^****^q < 0.0001), post-exertional malaise (PEM; *F*_2.81, 132_ = 28.3, ^****^q < 0.0001), and orthostatic intolerance (OI; *F*_2.79, 132_ = 12.4, ^****^q < 0.0001). From baseline to timepoint 3, the average difference in fatigue was 1.34 (95% CI: 0.55 to 2.14); disturbed sleep was 2.22 (95% CI: 1.14 to 3.30); PEM was 3.34 (95% CI: 2.31 to 4.37); and OI was 1.79 (95% CI: 0.95 to 2.63). Post-hoc Dunnett’s tests confirmed significant reduction in fatigue from baseline to time points 1, 2, and 3 (^****^q < 0.0001, ^****^q < 0.0001, and ^***^q = 0.0007, respectively). Similarly, disturbed sleep improved significantly from baseline to each time point (^***^q = 0.0009, ^****^q < 0.0001, and ^****^q < 0.0001), as did PEM (^****^q < 0.0001, ^****^q < 0.0001, and ^****^q < 0.0001) and OI (^***^q = 0.0009, ^***^q = 0.0002, and ^****^q < 0.0001). These results indicated that rapamycin therapy produced sustained and clinically meaningful improvements in symptoms central to ME/CFS, particularly fatigue, PEM, sleep disturbance, and orthostatic intolerance.

### Multidimensional fatigue inventory

Regarding the MFI (Fig. 1D), patients reported a statistically significant improvement across all fatigue domains: general fatigue (*F*_2.77, 131_ = 6.87, ^****^q < 0.0001), physical fatigue (*F*_2.86, 138_ = 4.50, ^***^q = 0.0008), reduced activity (*F*_2.50, 119_ = 9.71, ^****^q < 0.0001), reduced motivation (*F*_2.22, 107_ = 6.00, ^***^q = 0.0004), mental fatigue (*F*_2.79, 134_ = 6.92, ^****^q < 0.0001), and the aggregate (*F*_2.60, 127_ = 13.9, ^****^q < 0.0001). Especially in the latter, there was a significant 7.35-point average difference (95% CI: 3.79 to 10.9) from baseline to timepoint 3. Dunnett’s multiple comparisons test revealed significant improvements from baseline to time points 1, 2, and 3 in the aggregate (^***^q = 0.0009, ^****^q < 0.0001, and ^****^q < 0.0001). Stratifying by MFI domain, there were significant improvements from baseline to each timepoint in general fatigue (^**^q = 0.008, ^**^q = 0.004, and ^*^q = 0.04), physical fatigue (^*^q = 0.02, ^*^q = 0.03, and ^**^q = 0.008), reduced activity (^**^q = 0.002, ^***^q = 0.0005, and ^***^q = 0.0007), and mental fatigue (^*^q = 0.02, ^**^q = 0.001, and ^**^q = 0.005). With respect to reduced motivation, there was improvement from baseline to timepoints 2 and 3 only (^***^q = 0.0005 and ^***^q = 0.0005).

### RAND 36-item short form

On average, patients generally reported significantly higher scores over time for each SF-36 subscale (Fig. 1E), including physical functioning (*F*_2.56, 125_ = 4.28, ^**^q = 0.0013), role limitations due to physical health (*F*_2.68, 130_ = 5.09, ^***^q = 0.0005), energy and fatigue (*F*_2.71, 132_ = 12.6, ^****^q < 0.0001), emotional well-being (*F*_2.73, 133_ = 3.94, ^**^q = 0.0014), social functioning (*F*_2.44, 119_ = 4.13, ^**^q = 0.0014), bodily pain (*F*_2.49, 121_ = 2.18, ^*^q = 0.01), and general health (*F*_2.24, 109_ = 4.10, ^**^q = 0.002). However, this was not observed in the role limitations due to emotional problems subscale (*F*_2.75, 141_ = 0.40, *q* = 0.07 [ns]). Nonetheless, this is consistent with the leading theory that ME/CFS is not psychosomatic in etiology [47]. With respect to the aggregate scores—the Physical Component Score (PCS) and the Mental Component Score (MCS)—there was significant improvement in both the former (*F*_2.74, 153_ = 3.96, ^**^q = 0.0014) and the latter (*F*_2.61, 145_ = 3.05, ^**^q = 0.004). Post-hoc testing with Dunnett’s test revealed significant improvements from baseline to timepoint 1 in the following subscales: role limitations due to physical health, energy/fatigue, emotional well-being, and general health (^**^q = 0.006, ^***^q = 0.0006, ^*^q = 0.04, ^**^q = 0.005, respectively). Similar improvements were also found at time point 2 in the role limitations due to physical health, energy and fatigue, emotional well-being, social functioning subscales (^*^q = 0.01, ^****^q < 0.0001, ^*^q = 0.02, ^*^q = 0.01) and in PCS and MCS (^*^q = 0.02 and ^*^q = 0.03). Significant increases in physical fatigue, role limitations due to physical health, energy and fatigue, emotional well-being, social functioning, and general health were also found at time point 3 (^*^q = 0.03, ^*^q = 0.04, ^***^q = 0.00095, ^*^q = 0.049, ^*^q = 0.03, ^*^q = 0.01). PCS also had significant improvements from baseline to timepoint 3 (^*^q = 0.02).

Collectively, our results suggest that 90 days of low-dose rapamycin treatment significantly improved ME/CFS symptoms, based on the SSS inventory, MFI, and SF-36 scores.

### Effect of Rapamycin on the improvement of autophagy in trial participants

As an mTOR inhibitor, rapamycin is expected to improve autophagy flux [48] in ME/CFS patients, and increasing levels of serum BECLIN-1 are considered reliable markers for the overall improvement of autophagy flux [49, 50].

Interestingly, we observed a significant upregulation of BECLIN-1 (log2 transformed; *F*_1.46, 57_ = 6.5, ^***^q = 0.0009) in most ME/CFS cases (Fig. 2A). Dunnett’s multiple comparisons test further revealed significant increases of 1.4- and 1.5-fold from baseline to timepoints 2 (^**^q = 0.008) and 3 (^*^q = 0.02), respectively.

Our previous study [10] identified elevated serum concentrations of multiple phosphorylated ATG13 species as a potential pathogenic mechanism of ME/CFS. However, the antibody used (targeting Ser344) lacked specificity for detecting the mTOR-dependent phosphorylation of ATG13 in human samples, and no commercially available antibody was available. To accurately assess this, we developed a novel pSer258-ATG13 antibody to optimize the detection of pSer258-ATG13 by ELISA (Supplementary Fig. 4). As a quality-control (QC) validation, we first performed a series of dose- and time-sensitive ELISA analyses (Supplementary Fig. 4B) to identify the optimum dose and time of pSer258-ATG13 detection by using purified standards. After that, we performed a standard curve analysis (Supplementary Fig. 4C), which generated a strong linear correlation of doses with signal intensity (the squared regression coefficient = goodness of curve fit = r^2^ = 0.93;***p < 0.0001). Interestingly, we observed a significant reduction in circulating pSer258-ATG13 concentrations following initiation of low-dose rapamycin therapy (F_1.6, 54.2_ = 22.1, ^****^q < 0.0001; Fig. 2B). Post-hoc analyses demonstrated a consistent decrease in pSer258-ATG13 levels from baseline across all time points: a one-fold reduction at timepoint 1 (^**^q = 0.005), a 0.8-fold reduction at timepoint 2 (^*^q = 0.02), and a more than two-fold reduction by timepoint 3 (^****^q < 0.0001). These findings suggest that low-dose rapamycin effectively suppresses mTORC1-mediated phosphorylation of ATG13 over time in patients with ME/CFS.

Next, we performed a series of correlation analyses to evaluate the relationship between the changes in autophagy markers and fatigue-related symptom scores (Fig. 2C-F). Out of the correlation coefficients (Fig. 2E), increases in BECLIN-1 levels from baseline to T3 were positively correlated with improvements in several clinical outcome measures, including the BAS (*r*_*s*_ = 0.44, ^**^p = 0.01) and multiple subscales of the SF-36: physical functioning (PF; *r*_*s*_ = 0.40, ^*^p = 0.03), energy and fatigue (VT; *r*_*s*_ = 0.41, ^*^p = 0.02), emotional well-being (MH; *r*_*s*_ = 0.47, ^**^p = 0.01), general health (GH; *r*_*s*_ = 0.42, ^*^p = 0.02), and Mental Component Summary (MCS; *r*_*s*_ = 0.38, ^*^p = 0.04). Linear regression analysis suggests a strong inverse relationship between the BECLIN-1 levels and SF-36 energy/fatigue (VT) subscale scores (Fig. 2C).

Conversely, BECLIN-1 levels were negatively correlated with several subdomains of the MFI including physical fatigue (*r*_*s*_ = − 0.48, ^**^p = 0.006) and reduced activity (*r*_*s*_ = − 0.59, ^***^p = 0.0006), as well as the SSS symptoms disturbed sleep (*r*_*s*_ = − 0.47, ^**^p = 0.01) and orthostatic intolerance (*r*_*s*_ = − 0.59, ^***^p = 0.0006).

In contrast to BECLIN-1, when evaluating pSer258-ATG13, we observed a single, significant positive correlation between the reduction in biomarker and improvement in the SSS PEM symptoms (*r*_*s*_ = 0.42, ^*^p = 0.03; Fig. 2D). However, no significant correlations were observed between pSer258-ATG13 and the BAS, MFI domains, other SSS symptoms, and any SF-36 subscale or aggregate score (Fig. 2F).

### Low-dose Rapamycin does not affect clinical safety lab results

mTOR is a central regulator of key cellular processes, including metabolism and autophagy. Rapamycin, a selective mTOR inhibitor, is hypothesized to enhance autophagic activity by promoting sequestration of phosphorylated ATG13. However, mTOR inhibition may also suppress essential metabolic functions such as lipolysis and protein catabolism. Prior studies in both animal models and humans have shown that high-dose daily rapamycin administration can lead to adverse metabolic effects, including hypercholesterolemia, anemia, thrombocytopenia, and insulin resistance. In contrast, using our low-dose weekly administration protocol, we observed no adverse changes in safety laboratory parameters in our cohort from baseline through time point 3 (Supplementary Figs. 5 A–6B). This absence of laboratory abnormalities, combined with the low incidence of reported adverse events, highlights the favorable safety profile of weekly low-dose rapamycin in patients with ME/CFS.

Taken together, our pilot study demonstrated that low-dose rapamycin (6 mg/week) significantly ameliorated the clinical symptoms of fatigue and was well-tolerated in this relatively large ME/CFS cohort. Importantly, these findings support a mechanistic role of mTOR suppression in the restoration of autophagy. Rapamycin treatment is associated with a marked reduction in pSer258-ATG13, a key indicator of impaired autophagy. This reduction was correlated with an increase in BECLIN-1 expression, consistent with enhanced autophagy flux.

### A proposed machine learning model to predict responder subtype for future analyses

Using the procedure described in the Methods section, we were able to train a multinomial logistic regression machine learning model in which participants who did not complete the study, but had completed the ramp period, could be stratified based on previously verified and novel MCIDs for the different questionnaires.

Missing data (tabulated in Supplementary Table 1) were imputed using MICE with 10 maximum iterations. Data imputation with k-nearest neighbors (KNN) was also considered with k = 5 and k = 10. Post-imputation, observational analysis indicated that the mean and standard deviation across features were similar among the imputation methods, with negligible differences in summary statistics. MICE was ultimately selected for its superiority regarding complex data distributions and data potentially missing not at random, thus making it a more robust option.

The initial model was trained on the differentials of the 40 completers, which were subset into 24 partial responders, 10 responders, and 6 non-responders. Overall, the model had an accuracy of 95%, with the non-responder class consistently achieving precision, recall, and F1-score of 0.83, the partial responder class achieving 0.96, and the responder class achieving 1.00 across the same metrics. Confusion matrix (Supplementary Fig. 7A) and calibration curves (Supplementary Fig. 7B) were computed accordingly. Stratified, 5-fold cross-validation was performed with accuracy 0.73 (SD: 0.15). To improve stability, noise, and generalization, RepeatedStratifiedKFold was used with 3 splits and 5 repeats, and the accuracy was averaged to be 0.70 (SD: 0.12). Understandably, a larger sample size for the non-responder class would improve model performance. Nonetheless, in its initial, exploratory phase, the model is well-balanced.

All feature coefficients were extracted from the initial model, and odds ratios were computed (Supplementary Table 2). However, this approach yielded infinite 95% confidence interval bounds for several odds ratios, suggesting model instability. To address this, a two-step approach was implemented, starting with L1 regularization, employed to select variables across the three classes of responders most likely to stabilize the model and influence predictors, followed by model refinement with those selected variables. The four selected features were the MFI reduced motivation and aggregate scores as well as the SF-36 SF and PCS aggregate scores, and the coefficients were reported in Supplementary Table 3. For non-responders, the feature that greatly influences its classification is the MFI aggregate, where the odds ratio of $\:{e}^{\beta\:(MFI\:agg.)}$ = 3.87 (95% CI: 2.23 to 6.71; $\:{\beta\:}_{MFI\:agg.}$=1.35 [95% CI: 0.80 to 1.90]) suggests higher aggregate scores are significantly associated with an approximately four-fold increased odds of non-responder stratification relative to other classes, after adjusting for other predictors. Conversely, for responders, with the MFI aggregate, the odds ratio of 0.19 (CI: 0.11 to 0.33; $\:{\beta\:}_{MFI\:agg.}$= -1.65 [95% CI: -2.21 to -1.10]) suggests higher aggregate scores are significantly associated with an approximate 80% decrease in classification as a responder relative to other classes, adjusting for other features. However, higher SF-36 social functioning scores increased the odds (2.33, 95% CI: 1.52 to 3.57) of being associated as a responder.

For partial responders, all odds ratios included 1.00 in the 95% confidence interval, suggestive of no significant predictors for this class. It is possible that there is large feature overlap with non-responders and responders, thus limiting the predictive value of the model for this class. This limitation, however, is representative of the clinical complexity of a third predictive class compared to a simple binary classification. Some participants greater differences in one questionnaire, but not so much in other questionnaires, which contributed to the heterogeneity of responses. Given how a large number of participants were stratified as a partial responder, this multinomial logistic model meets the need for three classes, and adding features with definitive measures of improvement may make the model more robust.

The refined model with the four selected variables maintained strong performance with 3 × 5 RepeatedStratifiedKFold cross-validation yielding an overall accuracy of 0.71 (SD: 0.17). Classification report analysis revealed an average accuracy of 0.88, with confusion matrix (Supplementary Fig. 8A) and calibration curves (Supplementary Fig. 8B) calculated. The precision, recall, and F-1 scores for all classes ranged from 0.82 to 0.91, suggestive of balanced stratification. Although the original, full-feature model had greater accuracy at 95%, there is potential for overfitting and multicollinearity; thus, while the refined model has slightly lower performance, it is certainly more robust, especially when used towards stratifying non-completers.

Finally, this model was applied to predict the responder status of the non-completers (those with baseline and timepoint 1 completed at the minimum, but did not reach T = 3 dosing). 12 participants were classified as non-responders, 11 as partial responders, and 7 as responders. Among all of the 70 participants, there was a final total of 18 non-responders, 35 partial responders, and 17 responders based on the decision tree classifier for completers and model predictions for non-completers. We have demonstrated that the model has high overall fidelity, and future directions with more edge cases would improve the accuracy of the model in the partition of partial responders.

## Discussion

Myalgic encephalomyelitis/chronic fatigue syndrome (ME/CFS) is a debilitating, multisystem chronic illness with no approved treatments or objective diagnostic biomarkers. Our prior research in both human subjects and a mechanistically informed animal model identified chronic mTOR activation and subsequent autophagy impairment as potential drivers of ME/CFS pathogenesis, particularly in relation to post-exertional malaise (PEM), a hallmark symptom of the disease. Leveraging this mechanistic insight, the present study assessed the clinical efficacy of weekly low-dose rapamycin in improving disease-defining symptoms in patients with ME/CFS. Importantly, we also developed two blood-based molecular assays targeting BECLIN-1 and pSer258-ATG13 as objective biomarkers of autophagy function, which may facilitate future stratification of responders and guide precision therapeutic approaches in this highly heterogeneous population.

Rapamycin was well tolerated in this cohort, with a low incidence of adverse events, most of which were transient and nonserious. No significant changes were observed in safety laboratory parameters across the study duration, and there was no evidence of increased susceptibility to infections, addressing concerns regarding immunosuppression at this dosing schedule. All adverse events (AEs) leading to study discontinuation are shown in Table 1. Interestingly, an inability to participate due to financial constraints, combined with dropouts due to “no perceived clinical benefit”, accounted for over 67% of all study dropouts. Other reported AEs, including GI complaints and diarrhea (most reported), appear transient, beginning late in the ramp phase and then subsiding once the patient is established on the study dose. Taken together, these findings support the safety of once-weekly low-dose rapamycin in ME/CFS and justify further investigation in larger controlled trials.

### Rapamycin improves physical and mental fatigue symptoms in ME/CFS

Several lines of this manuscript demonstrate that rapamycin treatment effectively ameliorated fatigue-related symptoms in ME/CFS subjects. *First*, BAS analysis revealed a strong and significant improvement in daily physical activity in all participants from the BSL to T3 stages. *Second*, symptom analysis from the SSS inventory indicated a strong and significant recovery in overall fatigue, disturbed sleep, post-exertional malaise, and orthostatic intolerance among ME/CFS patients. *Third*, evaluations of different fatigue parameters of MFI also revealed a significant restoration of health deficits, such as general fatigue, reduced motivation, physical fatigue, mental fatigue, and reduced activity. Finally, the SF-36 score analysis revealed a strong recovery of social functioning and physical and emotional health. Our analysis was unbiased, based on age, sex, body weight, and disease duration. The effect of rapamycin on the overall metabolism of sugars, lipids, and cholesterol was monitored in all patients during each study time point; however, no significant differences were observed.

### Rapamycin may exhibit a significant improvement of autophagy flux in ME/CFS subjects

Rapamycin inhibits mTOR and is therefore expected to repair the mTOR-driven inhibition of autophagy. To investigate the effect of rapamycin on the improvement of the overall autophagy flux, we performed a quantitative ELISA using the pan-autophagy marker BECLIN-1. Interestingly, rapamycin significantly increased the plasma levels of BECLIN-1 at the T2 and T3 stages compared to BSL, suggesting that rapamycin may improve the overall autophagy flux in ME/CFS subjects. To better understand the effect of rapamycin on autophagy, we categorized the cohort into three different groups based on the recovery score. The “responder” group (*N* = 17) with the most significant fatigue recovery based on MFI and SF-36 evaluations, displayed the strongest response of BECLIN-1 upregulation from BSL to T2 to T3 stages. The “partial responder” (*N* = 18) group and “non-responder” (*N* = 5) groups did not show any significant upregulation in BECLIN-1 levels. Interestingly, while evaluating the plasma levels of BECLIN-1 in the viral-onset (*N* = 36 ME/CFS cohort), we observed a robust upregulation of BECLIN-1 from BSL to T2 and T3 stages, suggesting that long-term treatment with rapamycin improves overall autophagy in post-infectious ME/CFS subjects. Next, we studied whether the upregulation of BECLIN-1 was correlated with the alleviation of fatigue symptoms. Our correlation analysis suggests a significant correlation between BECLIN-1 upregulation and the alleviation of symptoms, as measured by the MFI total score and two key sub-scores provided by the instrument, including physical fatigue and reduced motivation. Similarly, the correlation was also very strong in the viral-onset group, suggesting that rapamycin treatment could be an effective intervention in a subset of patients with documented beta herpes virus onset and/or reactivation syndrome.

ATG13 is considered a direct target of mTOR. Upon phosphorylation at its serine 258 residue, ATG13 becomes inactivated and aborts the initiation complex of autophagosome formation, resulting in the impairment of cellular autophagy. Our recent animal model study also showed that the chronic activation of mTOR inhibits ATG13 in inducing ME/CFS-like post-exertional malaise. To determine the clinical relevance of pSer258-ATG13 inactivation in ME/CFS subjects, we developed a Serine258P-specific ATG13 antibody that was used in a highly sensitive indirect ELISA. Interestingly, our pSer258-ATG13 ELISA study demonstrated a significant downregulation of pSer258-ATG13 from the BSL (*N* = 40) to the T1(*N* = 36), T2 (*N* = 38), and T3 (*N* = 32)stages in *N* = 40 ME/CFS patients. While correlating the pSer258-ATG13 level with clinical symptoms of fatigue, it was found that the downregulation of the pSer258-ATG13 level was positively correlated with the alleviation of the reduced activity domain in the MFI. Similarly, we observed strong downregulation in the plasma levels of pSer258-ATG13 in the rapamycin-treated post-infectious ME/CFS cohort. The downregulation was significant from BSL to all three stages, with the maximum reduction at the T3 stage. However, no significant correlation was observed between pSer258-ATG13 downregulation and alleviated fatigue symptoms in the viral-onset ME/CFS group, mainly because of a dramatic decrease in pSer258-ATG13 at the T3 stage. The dynamic range of plasma pSer258-ATG13 levels may be affected by inter-assay variability; however, this variability was successfully mitigated through multiple repetitions of the assay by several researchers following a standardized operating protocol, as detailed in the methods section.

The detection of autophagy-related proteins in extracellular environment [51] has been demonstrated through analysis of the autophagy-associated secretome [52]. Key autophagy-related proteins including ATG5 [53], ATG7 [54], ATG13 [11], as well as other regulatory proteins [55]such as Beclin-1, LC3B, and VAMP7 are integral constituents of this secretome. The increased detection of pSer258-ATG13 in ME/CFS subjects, along with its reduction following rapamycin treatment, may be attributed to evidence suggesting that mTOR activation [56] contributes to the secretion of exosomes containing autophagy-related proteins in neurodegenerative conditions [57]. Furthermore, administration of the mTOR inhibitor rapamycin has been shown to significantly decrease the release of these exosomes [58]. A recent report [59] also suggests that ME/CFS pathogenesis are also involved with the increased cellular permeability, which may be responsible for the elevated levels of intracellular proteins. The physiological detection limits of similar ATG proteins, including ATG5 [53] and ATG7 [54], have been assessed by other research groups. Reported bioavailability levels of these proteins in plasma samples are comparable to the concentration range detected in our study. A few essential steps in our next phase of the trial will be running LC/MS study in plasma samples to quantify phospho ATG13 and the comparative analyses between intracellular (PBMC lysate) and extracellular levels (Plasma) of ATG13 to understand the physiological regulation of ATG13.

### Significance, limitations, challenges, and future directions

This study represents one of the first biomarker-targeted clinical trials of ME/CFS. To date, no definitive molecular mechanism has been established for the pathogenesis of ME/CFS, and no prior treatment trials have focused on mechanistic targets. Our findings provide the first clinical evidence linking mTOR activation and impaired autophagy to the ME/CFS pathophysiology. Notably, the observed clinical improvement in fatigue was paralleled by molecular evidence of enhanced autophagy, including decreased pSer258-ATG13 and increased BECLIN-1 expression. This trial provides a critically important opportunity to study a subgroup of patients that can be stratified before enrollment based on a convenient molecular test that is likely to respond to a very safe, affordable, and FDA-approved agent.

However, this study had several limitations. Most notably, the absence of a placebo control group limited the ability to attribute clinical improvements to the intervention definitively. Functional outcomes and changes in symptoms were assessed solely through self-reported questionnaires without objective performance-based measures. Despite these limitations, the trial was designed to test a biomarker-driven hypothesis initially observed in *vivo*, namely that rapamycin modulates mTOR-mediated autophagy disruption. Given that each subject served as their own control, this within-subject design helps strengthen the internal validity of our findings. It is possible that markers like pSer258-ATG13 and BECLIN-1 could be used as future inclusion and exclusion criteria in randomized, placebo-controlled trials in addition to prognostic markers of response. Therefore, while we lack a placebo, our study demonstrates that a significant amount of valuable data can be generated from this observational trial design. Identifying specific mechanisms of treatment response (or lack thereof) using validated cohorts and objective biomarkers will be critical to de-risk larger controlled trials, given the known heterogeneity of this patient population.

Although a substantial number of participants discontinued the study before the final endpoint, we attribute this primarily to the pilot nature of the trial and limited funding, which prevented us from covering drug and laboratory costs for participants. A secondary reason for dropout was the lack of perceived clinical benefit or the initiation of alternative therapies that were incompatible with the study protocol. While the monthly cost out of pocket for each participant in this study ranged from $1–60 USD (based on insurance and other factors, including geographical location), the cost is reasonable compared to other proposed treatment protocols, including intravenous gamma globulin-IVIG, immune checkpoint inhibitors, B-cell depletion therapies, and antivirals. Our study shows, however, that all financial burdens related to study participation are paramount in our experience.

Another limitation of this study is the absence of a standardized generic formulation of the study drug. Prior research [60] has demonstrated considerable variability in the bioavailability of different generic sirolimus preparations, which may influence therapeutic response. Obtaining a matching placebo to the study drug formulation of choice also poses difficult choices for researchers, as placebos to various formulations vary significantly in availability and cost. Future studies should prioritize the use of a single, well-characterized formulation, whether compounded or generic, and incorporate pharmacogenetic profiling of drug-metabolizing enzymes, including CYP3A [61] and other cytochrome P450 [62] isoforms. Additionally, measuring peak and trough sirolimus levels may allow for sub-grouping patients based on metabolizer status and perhaps help hone individual doses for maximum clinical effect.

While trends in symptom improvement correlated with significant and positive changes in autophagy markers, statistical significance was not achieved in some analyses, likely because of the small sample size. Notably, subgroup analysis in patients with non-viral ME/CFS onset was limited by low enrollment, reducing the power to detect changes in pSer258-ATG13 expression. The detection of phosphorylated proteins in plasma presents significant challenges due to their instability and low concentrations. To address these issues, the use of phosphatase and protease inhibitor cocktails is recommended as outlined in the methods section. Plasma samples should be analyzed within 24 h of receipt, promptly stored in liquid nitrogen upon arrival, and care should be taken to minimize repeated freeze-thaw cycles. The detection range of our custom antibody conforms to the specifications provided by the manufacturer. Recognizing the intracellular origin of phosphorylated ATG13, the subsequent phase of our study will include measurement of pSer258ATG13 levels in lysed PBMCs. Preliminary data obtained are consistent with the findings presented in this report.

Enrollment in this study was limited to patients invited by the respective clinical PIs, resulting in a cohort with a clear post-infectious onset bias compared to other onset factors. Additionally, maintaining participant engagement throughout the trial posed some logistical challenges, perhaps due to this trial’s decentralized design. Accordingly, a comprehensive subgroup analysis will be conducted in future studies with an expanded sample size and focus enrollment on patients with multiple disease onset types, as this appears to be an essential factor.

To address some of the challenges outlined above, in February 2025, we launched an enhanced version of this trial with some key protocol adjustments and the inclusion of additional clinical PIs to expand our patient cohort. First, we developed a collaboration with a key strategic partner, AgelessRx^®^, to support centralized unformed study drug (and matching placebo) distribution in our decentralized study design. Second, we enhanced our biospecimen collection and biobanking protocol to obtain more diverse sample types for several high-throughput and high-resolution downstream applications, while also enhancing protocols for sample collection (mobile phlebotomy) and sample shipment. We have included wearable devices to objectively monitor clinical endpoints ranging from simple step count and “upright” time to more advanced monitoring, including heart rate variability, estimated VO2 max, and sleep score. In addition to the safety labs reported in this manuscript, which are centrally run by a commercial laboratory (Quest), our expanded phase includes a pregnancy test for females under 50 years old and quantitative sirolimus plasma levels at each time point. We have expanded our study observation time window from 90 days to 16 months to assess the long-term effects of this intervention. Finally, and to address the lack of a placebo, this enhanced trial will allow participants the option to enroll in a “placebo crossover study.” After completing the 90-day trial on rapamycin, participants will be randomized (1:1) to either continue the study drug or receive a placebo for an additional 60 days. At the end of the 60 days, or sooner if clinically indicated, all patients will be crossed back into the treatment group. Multiple patient-led research advocacy groups support such a design, particularly in repurposing studies like this, where the study drug is FDA-approved and available.

## Supplementary Information

Below is the link to the electronic supplementary material.

Supplementary Material 1

## Data Availability

De-identified questionnaire, biomarker, and analysis data have been uploaded to Mendeley and will be available following the link: DOI: 10.17632/k78d4cgcyx.1.

## Abbreviations

ME/CFS: Myalgic Encephalomyelitis/Chronic Fatigue Syndrome; rapamycin
mTOR: The mammalian target of Rapamycin; BECLIN-1
ATG13: Autophagy-related gene13-encoded protein
pSer258-ATG13: Serine-258 phosphorylated ATG13;
BAS: Bell Activity Scale
SSS: Specific Symptom Severity
PEM: Post-exertional malaise
OI: Orthostatic intolerance
MFI: Multidimensional Fatigue Inventory
SF-36I: RAND 36-Item Short Form Survey
PF: SF-36 physical functioning subscale
RP: SF-36 role limitations due to physical health subscale
RE: SF-36 role limitations due to emotional problems subscale
VT: SF-36 energy/fatigue subscale
MH: SF-36 emotional well-being subscale
SF: SF-36 social functioning subscale
BP: SF-36 bodily pain subscale
GH: SF-36 general health subscale
PCS: ;SF-36 Physical Component Score aggregate
MCS: SF-36 Mental Component Score aggregate
