# Supplementary material for: Low-dose rapamycin alleviates clinical symptoms of fatigue and PEM in ME/CFS patients via improvement of autophagy: a pilot study
Source: J Transl Med. 2025 Oct 21;23:1148. doi: 10.1186/s12967-025-07213-8 (PMC12538759; doi:10.1186/s12967-025-07213-8)
Supplement: Supplementary file 1 — Supplementary Material 1 [file 12967_2025_7213_MOESM1_ESM.docx]

**Supplementary File:**

**Low-Dose Rapamycin Alleviates Clinical Symptoms of Fatigue and PEM in ME/CFS Patients via Improvement of Autophagy: A Pilot Study**

Brian T. Ruan^1^, Sarojini Bulbule^2^, Brooke Gile^2^, Amy Reyes^2^, Bela Chheda^3^, Lucinda Bateman^4^, Jennifer Bell^4^, Brayden Yellman^4^, Maureen Hanson^2^, Stephanie Grach^5^, Jon Berner^6^, Daniel L. Peterson^7^, *David Kaufman ^3^, *Avik Roy^2,9^, *C. Gunnar Gottschalk^2,8^

**Affiliations:**

^1.^ Cornell EIND Center, Cornell University, Ithaca, NY

^2.^ Simmaron Research Inc. Research and Development Lab, University of Wisconsin-Milwaukee

^3.^ Centers for Complex Diseases, Palo Alto, CA and Seattle, Washington

^4.^ The Bateman Horne Center, Salt Lake City, UT

^5.^ The Mayo Clinic, Rochester, NY

^6.^ Woodinville Psychiatry, Woodinville, WA

^7.^ Sierra Internal Medicine, Incline Village, NV

^8.^ Simmaron Research Center for Translational Science, ICBI at Indiana University Methodist Hospital, Indianapolis, IN

^9.^ Milwaukee Institute for Drug Discovery, University of Wisconsin-Milwaukee, 2000 E Kenwood Blvd, Milwaukee, WI 53211

*Correspondence to

C. Gunnar Gottschalk PhD

Simmaron Research Center for Translational Science,

ICBI

Indiana University Methodist Hospital

Indianapolis, IN

[ggottschalk@simmaron.com](mailto:ggottschalk@simmaron.com)

David Kaufman, MD

Avik Roy, PhD

Simmaron Research & Development Laboratory,

Milwaukee Institute for Drug Discovery

University of Wisconsin-Milwaukee

2000 E Kenwood Blvd

SBIR suite # 320

Milwaukee, WI 53211

[aroy@simmaron.com](mailto:aroy@simmaron.com)

Center for Complex Diseases, CA

Center for Complex Diseases

Email: david@centerforcomplexdiseases.com

**Legends to Supplementary Figures:**

**Supplementary Figure 1. Normality testing results of residuals of mixed effect model.** QQ plots do not show any significant deviation which may be suggestive of violation of mixed effect model testing assumptions. (A) QQ plot of BAS, (B) QQ plot of SSS symptoms, (C) QQ plot of MFI, (D) QQ plot of SF-36 subscales and aggregates, (E) QQ plots of before and after log-transformation of BECLIN-1 concentration, (F) QQ plots of before and after log-transformation of pSer258ATG13 concentration.

**Supplement Figure 2. Low-dose rapamycin therapy schema and analysis of metabolic panel at different milestones of rapamycin treatment.** (A) A schema represents timepoints (T) of rapamycin treatment. T1 represents the first thirty-six (36) days of treatment with increasing doses of rapamycin starting from 1 (6 days) → 2 (6 days) → 3 (6 days) → 4 (6 days) → 5 (6 days) → 6 mg (6 days). After ramping period, the treatment enters T2 (60 days) phase with constant dosing of 6mg/week, then, finally, to T3 (90 days) phase. (**B**) The table itemized important documentation such as acquisitions of consent, detailed medical history, disease onset before the treatment began at BSL stage. Improvement of daily activity, fatigue, the overall quality of health, metabolic changes associated with lipid, sugar, and inflammatory metabolic processes were monitored at the end of each milestones.

**Supplement Figure 3. A flow-chart for the participants.** Response criteria was set based on the positive, no change, and/or negative reports of symptoms as reported on the SSS, MFI, and SF-36 inventories. Twenty (17) responders reported positive changes in selected symptoms from the SSS, all domains of the MFI, and the PCS of the SF-36. Nine (18) partial responders reported positive changes in some SSS symptoms and either the MFI or SF-36, whereas eleven (5) non-responders reported no change/negative reports in the SSS symptoms, MFI aggregate score, and SF-36 PCS aggregate.

**Supplementary Figure 4. Development of pSer258ATG13 ELISA.** (A) The peptide sequence was used as a substrate antigen to elicit immunogenic response in rabbit. Anti-sera were collected after 90 and 120 days of immunization and then affinity purified as per the protocol optimized by ThermoFisher Scientific. (B) Dose and time optimization of signal intensity were performed followed by the (C) derivation of standard curve. Results are mean ± SD after 3 different experiments.

**Supplementary Figure 5. The effect of rapamycin on blood cell count in ME/CFS subjects.** Quantifications of (A) WBC (10^3^/µL), (B) RBC (10^6^/µL), (C) hemoglobin (g/dL), (D) hematocrit (%), (E) platelet (10^3^/µL), and (F) hemoglobin A1C (%) were performed in all subjects at BSL, T1-T3 stages. No significant change was observed among different groups in any of these measurement criteria.

**Supplementary Figure 6. The effect of rapamycin on the metabolic status of ME/CFS subjects**. Glucose and lipid metabolic status of the cohort was evaluated by measuring (A) glucose (mg/dL, (B) creatine (mg/L), (C) triglyceride (mg/dl), (D) total cholesterol (mg/dL), (E) HDL (mg/dL), and (F) LDL (mg/dL) at different stages of rapamycin treatment. No significant change was observed among different groups in any of these measurement criteria.

**Supplementary Table 1. Summary of missing data for patient-reported outcome measures included in the machine learning model analysis.** Subscales with missing values were imputed using MICE. (A) All 70 completers and non-completers across 4 timepoints (BSL, T1, T2, and T3), totaling to 280 possible values; (B) only 40 completers across 4 timepoints, totaling to 160 possible values; (C) only 30 non-completers across 4 timepoints, totaling to 120 possible values

**Supplementary Figure 7. Confusion matrix and calibration curves summarizing the performance of the full feature multinomial logistic model.** (A) Confusion matrix shows almost fully correct categorization of classes, and (B) shows point estimates for calibration.

**Supplementary Table 2. Summary of regression coefficients (95% confidence interval) and odds ratios (95% confidence interval) for each feature in the model for non-responders only.** This table also includes the features with confidence intervals that were too large and non-interpretable (thus excluded from the table).

**Supplementary Figure 8. Confusion matrix and calibration curves summarizing the performance of the refined multinomial logistic model.** (A) Confusion matrix shows identical categorization of classes as with the full feature model, and (B) shows point estimates for calibration.

**Supplementary Table 3. Summary of regression coefficients (95% confidence interval) and odds ratios (95% confidence interval) for each selected feature in the model for each responder group.** Each coefficient is compared using the other classes as reference groups.

**
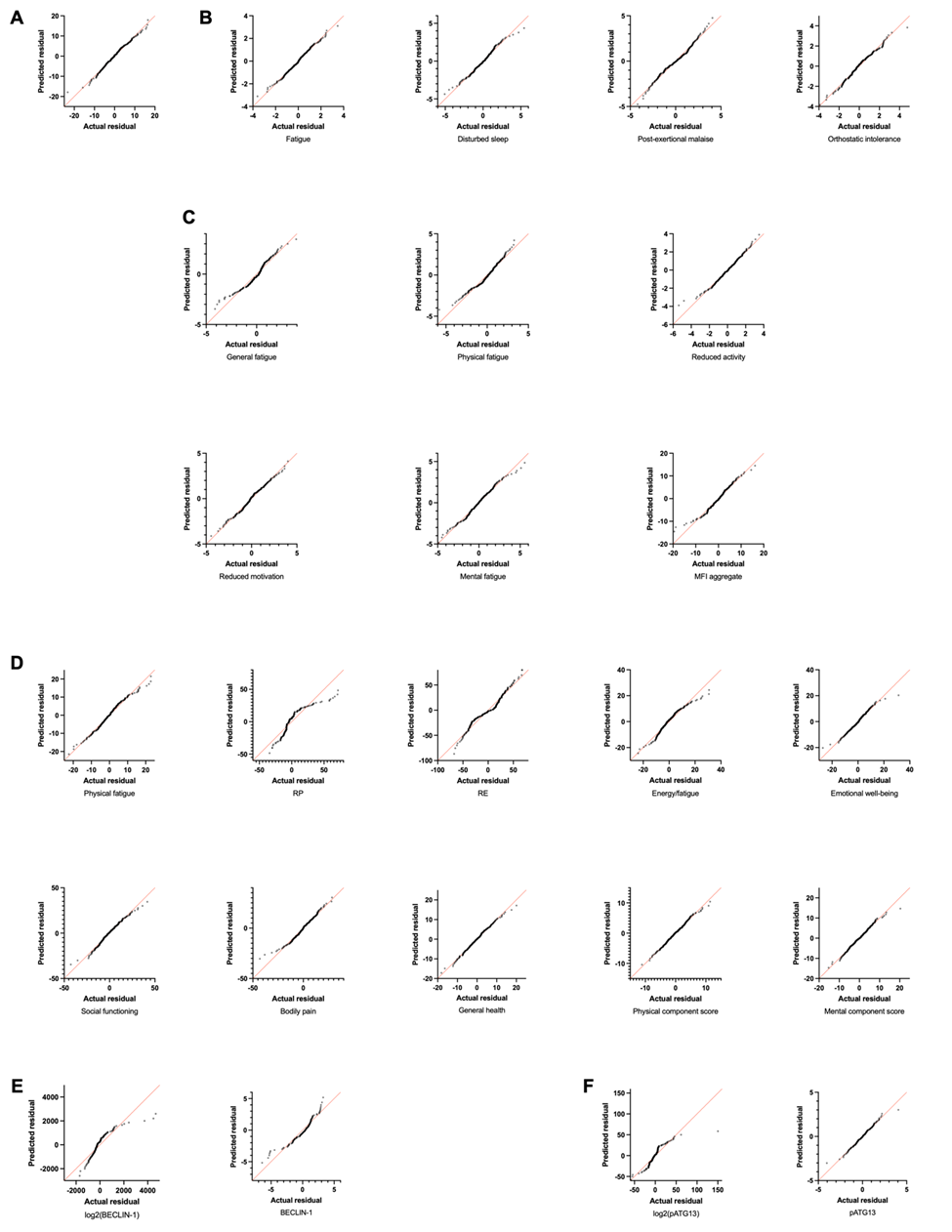
Supplementary Fig. 1.**


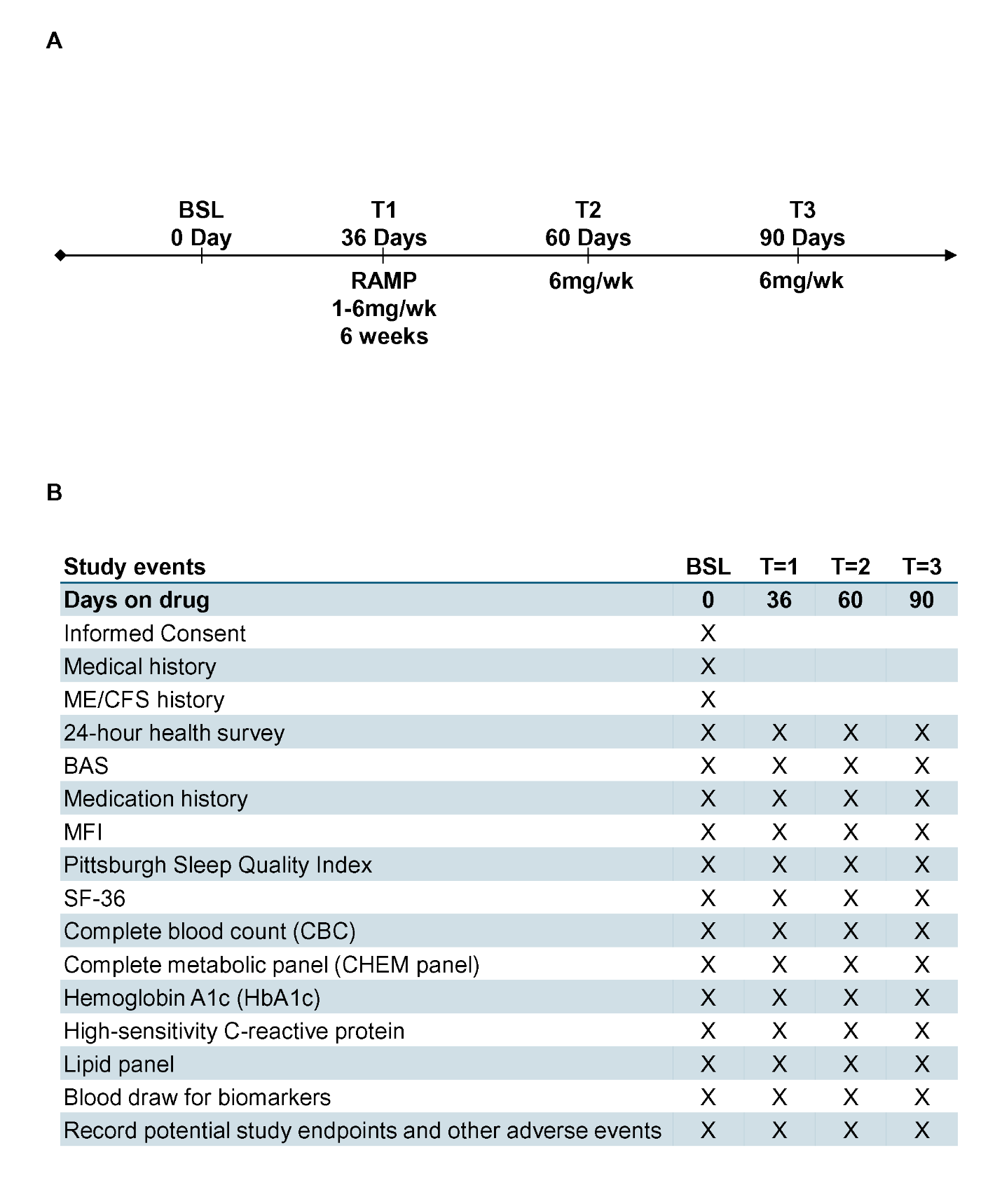


**Supplementary Fig. 2.**


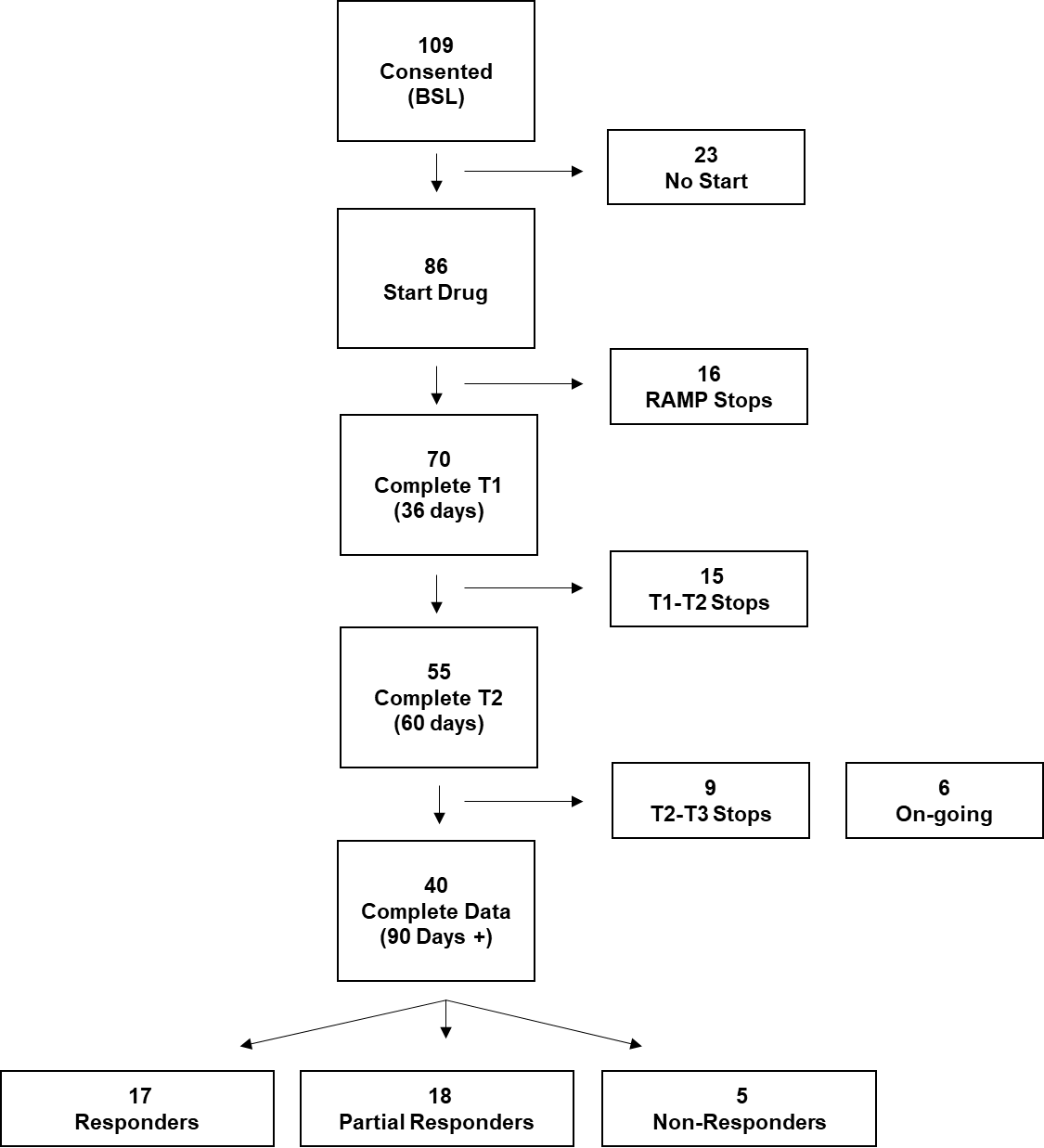


**Supplementary Fig. 3.**

**
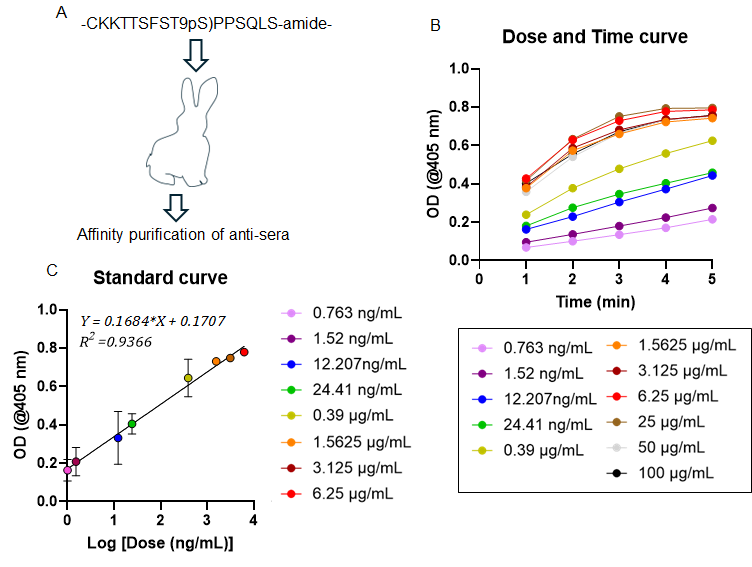
Supplementary Fig. 4.**

**
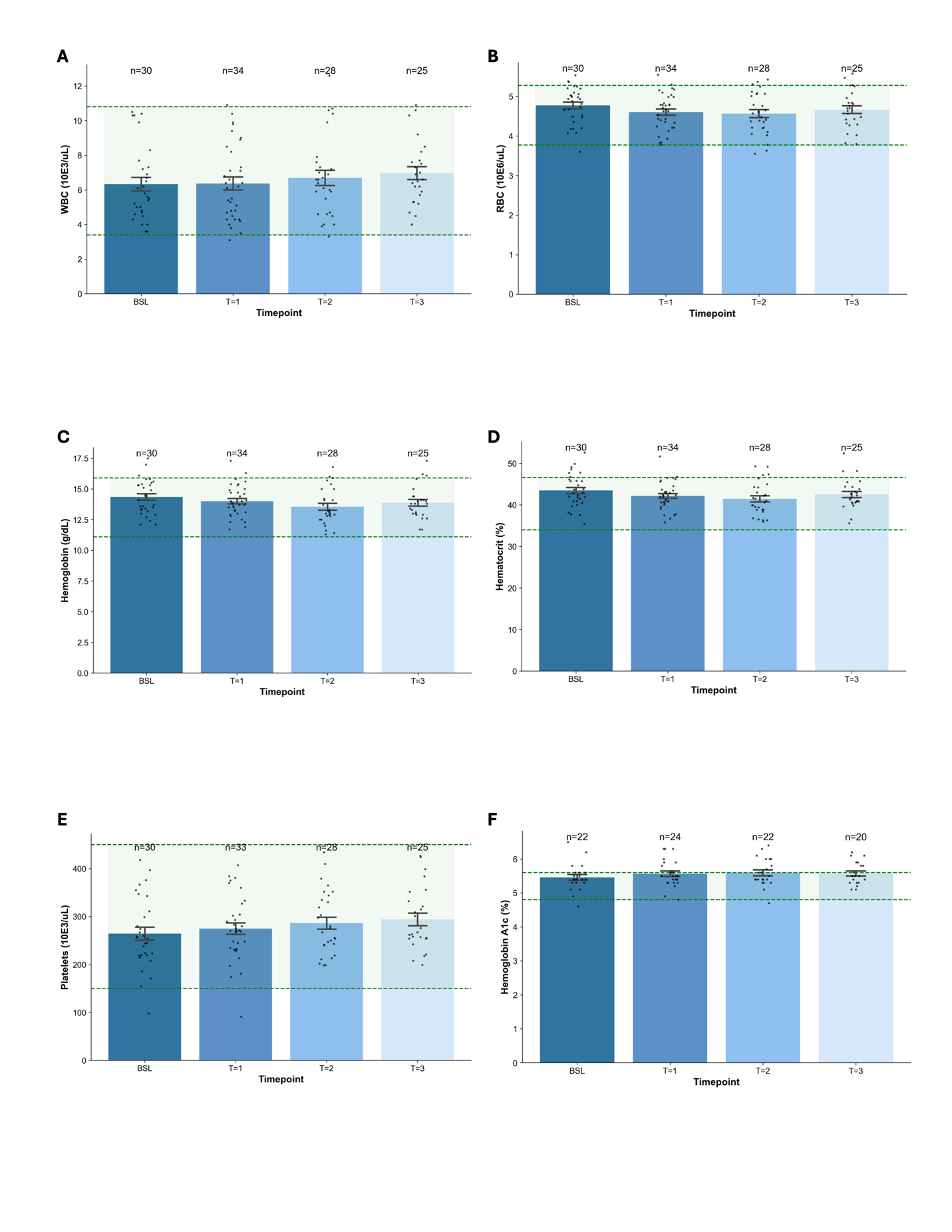
Supplementary Fig. 5.**


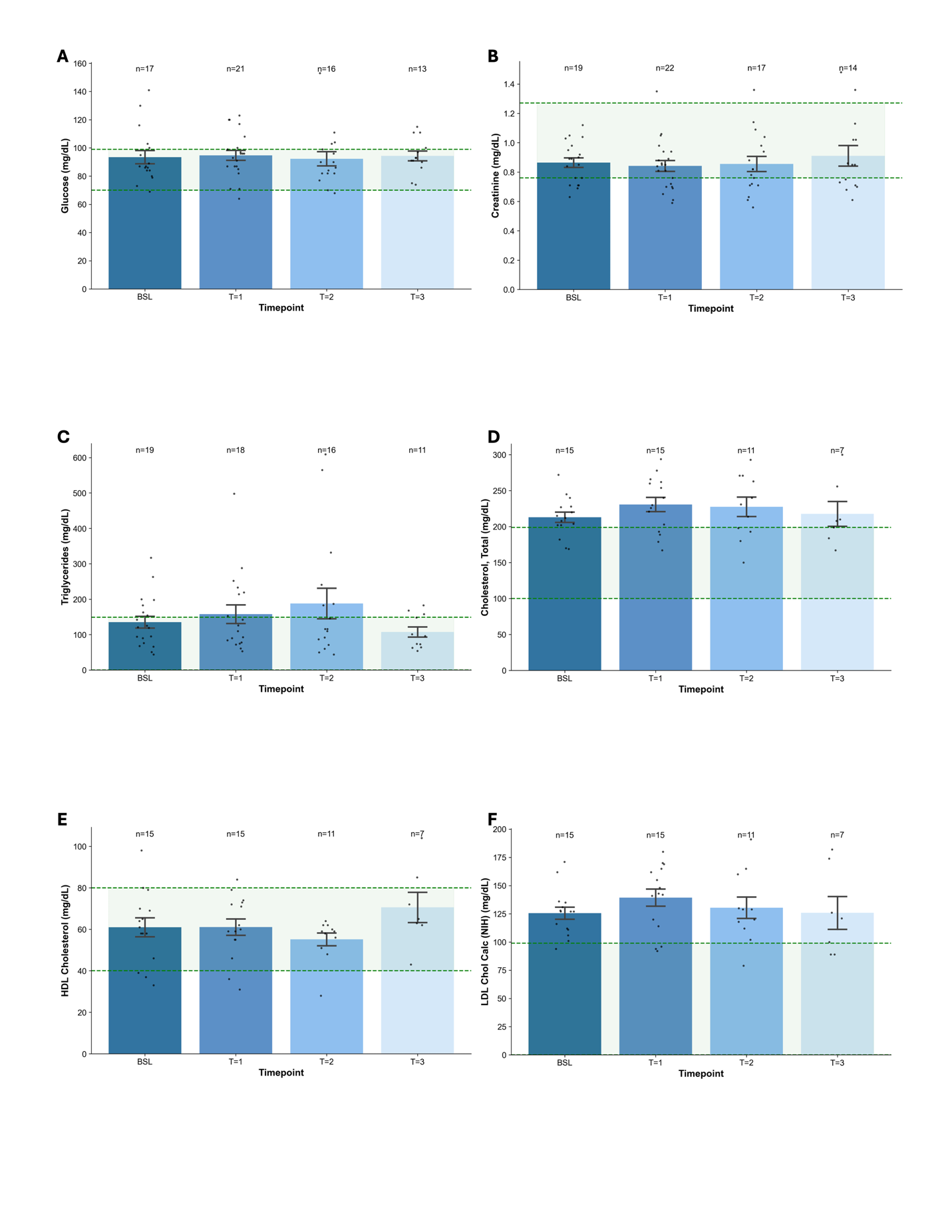


**Supplementary Fig. 6.**

**Supplementary Table 1.**

**
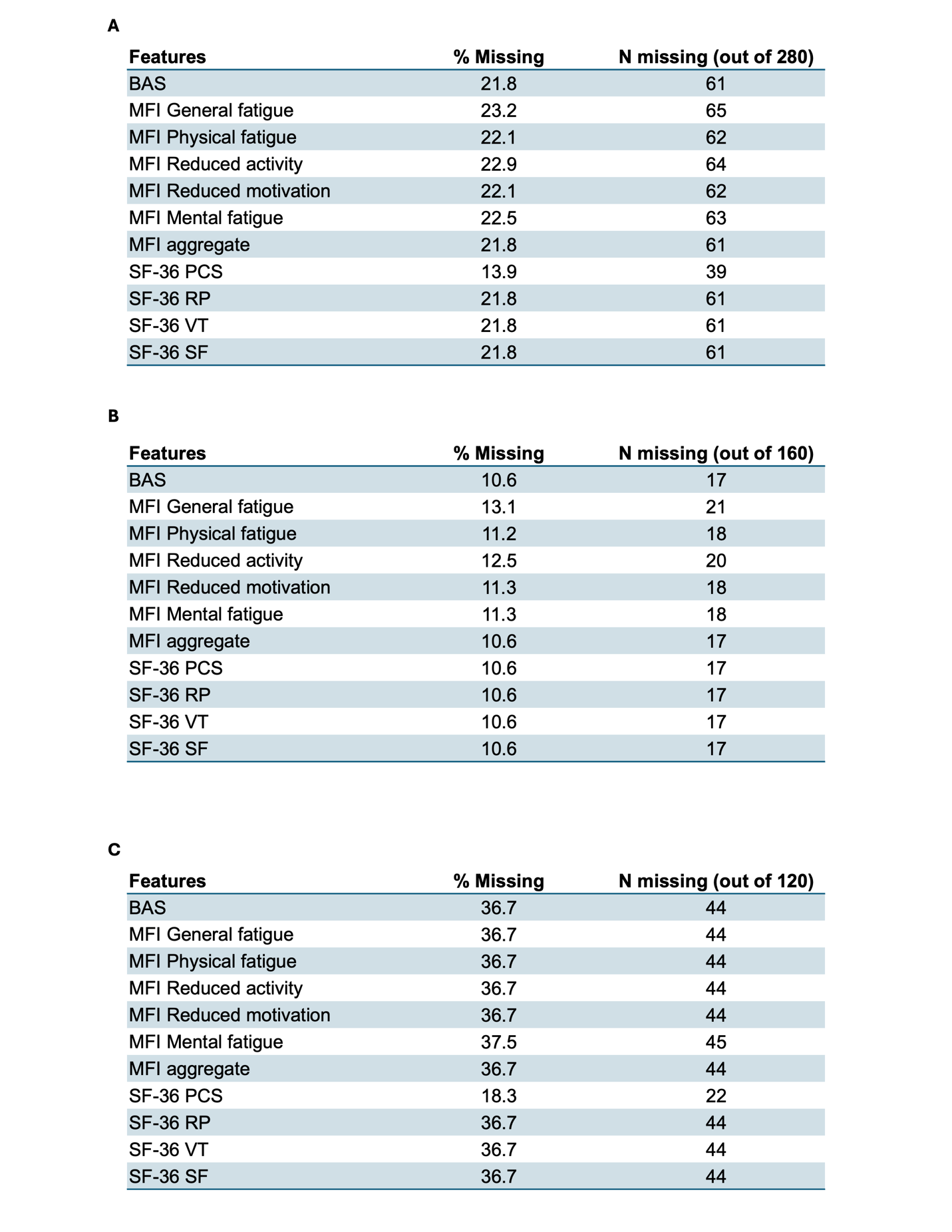
**


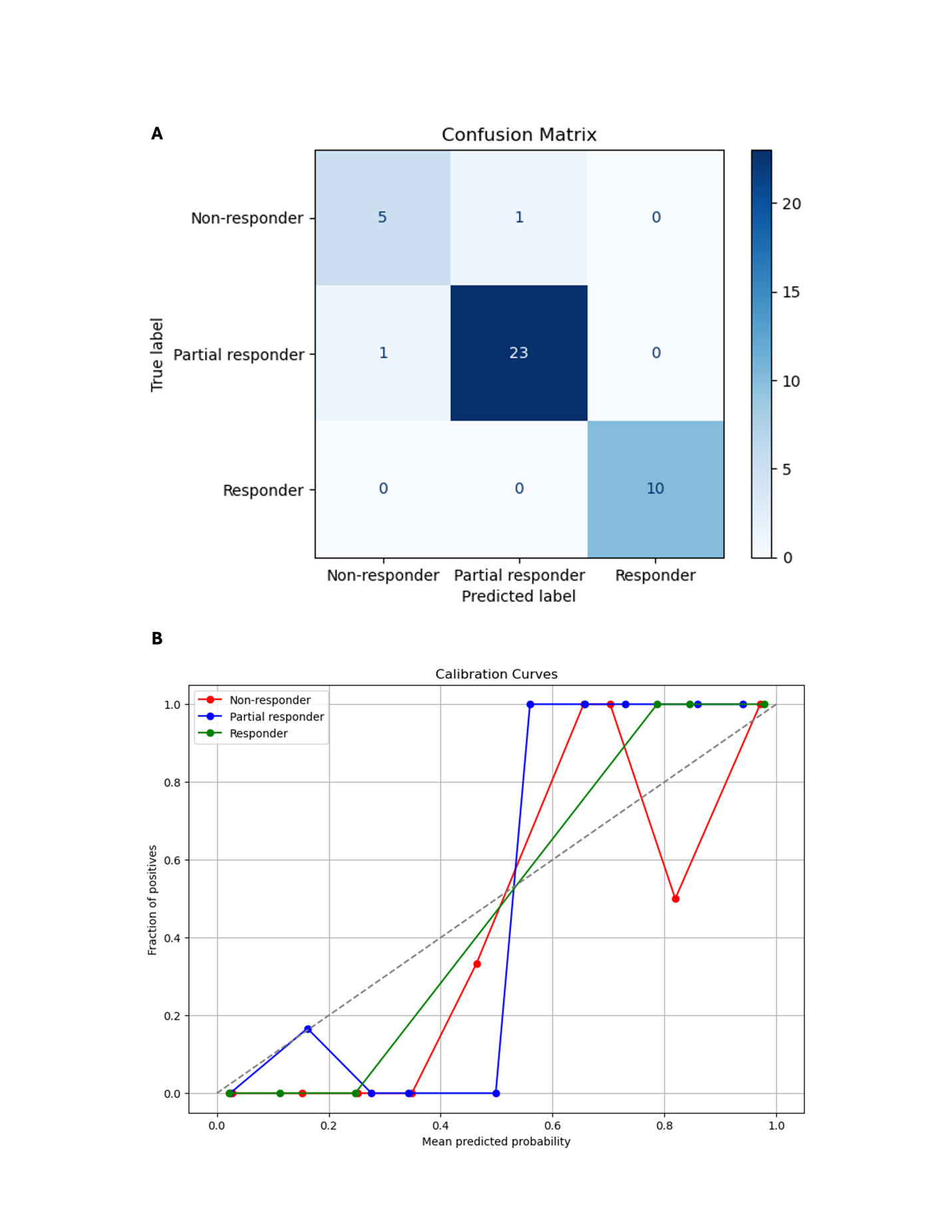


**Supplementary Fig. 7.**

**Supplementary Table 2.**

| Feature | Coefficients (95% CI) | Odds Ratios (95% CI) |
| --- | --- | --- |
| BAS | -0.26 (-0.28 to -0.23) | 0.77 (0.75 to 0.80) |
| MFI General Fatigue^†^ | 0.56 | 1.76 |
| MFI Physical Fatigue^†^ | 0.31 | 1.36 |
| MFI Reduced Activity^†^ | 0.72 | 2.06 |
| MFI Reduced Motivation^†^ | 0.81 | 2.24 |
| MFI Mental Fatigue^†^ | 0.21 | 1.23 |
| MFI aggregate^†^ | 0.88 | 2.41 |
| SF-36 PCS | -0.62 (-0.67 to -0.57) | 0.54 (0.51 to 0.57) |
| SF-36 RP | -0.37 (-0.88 to 0.14) | 0.69 (0.42 to 1.15) |
| SF-36 VT | -0.19 (-0.87 to 0.48) | 0.82 (0.42 to 1.62) |
| SF-36 SF | -0.68 (-1.07 to -0.29) | 0.51 (0.34 to 0.75) |

^†^ These features had exceedingly wide confidence intervals that were not reportable.


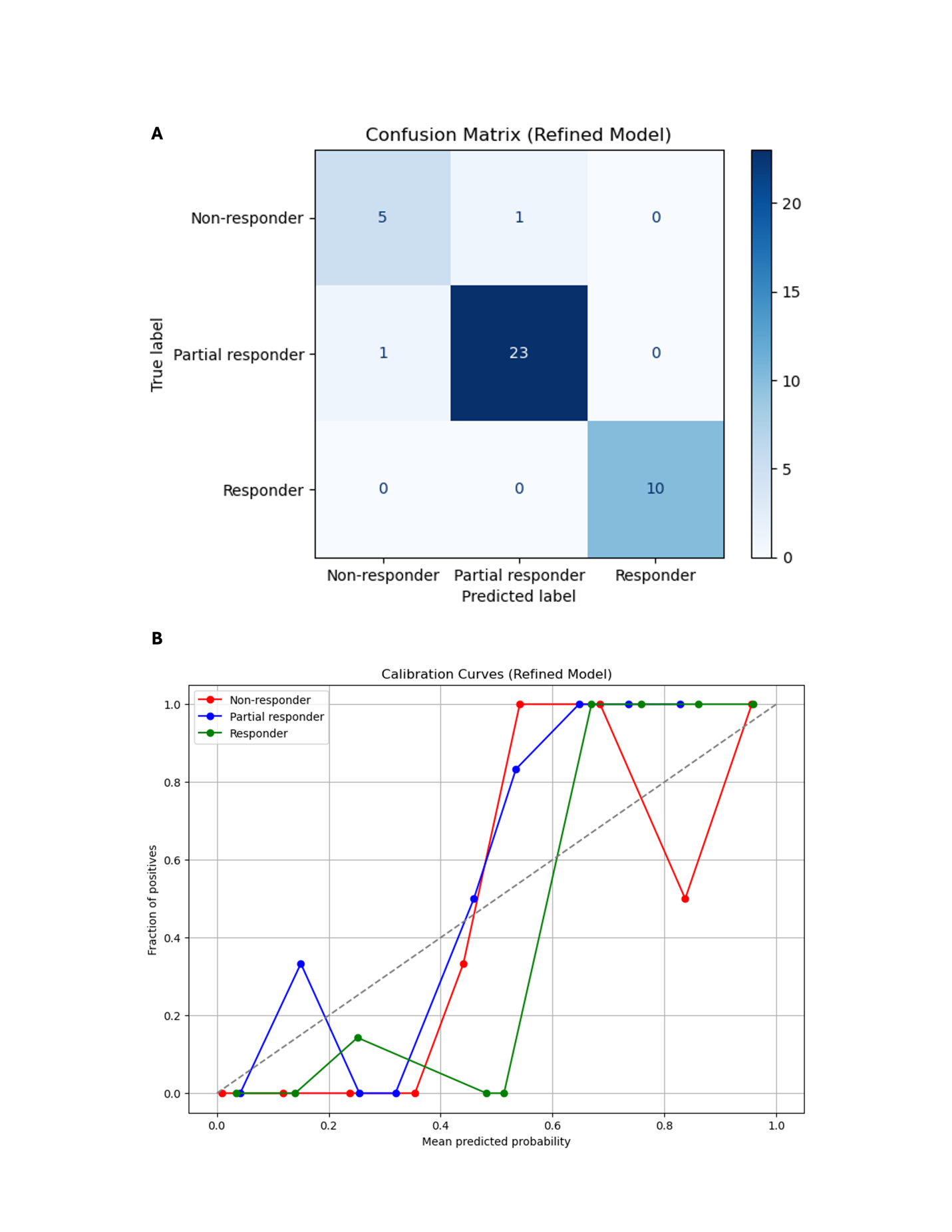


**Supplementary Fig. 8.**

**Supplementary Table 3.**

**A**

| Feature | Coefficients (95% CI) | Odds Ratios (95% CI) |
| --- | --- | --- |
| MFI Reduced Motivation | 0.81 (0.32 to 1.30) | 2.25 (1.37 to 3.68) |
| MFI aggregate | 1.35 (0.80 to 1.90) | 3.87 (2.23 to 6.71) |
| SF-36 PCS | -0.61 (-1.01 to -0.21) | 0.54 (0.36 to 0.81) |
| SF-36 SF | -0.87 (-1.29 to -0.44) | 0.42 (0.27 to 0.64) |

**B**

| Feature | Coefficients (95% CI) | Odds Ratios (95% CI) |
| --- | --- | --- |
| MFI Reduced Motivation | -0.30 (-0.80 to 0.19) | 0.74 (0.45 to 1.21) |
| MFI aggregate | 0.30 (-0.25 to 0.85) | 1.35 (0.78 to 2.35) |
| SF-36 PCS | 0.39 (-0.01 to 0.79) | 1.48 (0.99 to 2.21) |
| SF-36 SF | 0.02 (-0.40 to 0.45) | 1.02 (0.67 to 1.57) |

**C**

| Feature | Coefficients (95% CI) | Odds Ratios (95% CI) |
| --- | --- | --- |
| MFI Reduced Motivation | -0.51 (-1.00 to -0.01) | 0.60 (0.37 to 0.99) |
| MFI aggregate | -1.65 (-2.21 to -1.10) | 0.19 (0.11 to 0.33) |
| SF-36 PCS | 0.22 (-0.18 to 0.62) | 1.24 (0.83 to 1.85) |
| SF-36 SF | 0.85 (0.42 to 1.27) | 2.33 (1.52 to 3.57) |
